# Engineered CAR‐T cells targeting the non‐functional P2X purinoceptor 7 (P2X7) receptor as a novel treatment for ovarian cancer

**DOI:** 10.1002/cti2.1512

**Published:** 2024-05-23

**Authors:** Veronika Bandara, Victoria M Niktaras, Vasiliki J Willett, Hayley Chapman, Noor A Lokman, Anne M Macpherson, Silvana Napoli, Batjargal Gundsambuu, Jade Foeng, Timothy J Sadlon, Justin Coombs, Shaun R McColl, Simon C Barry, Martin K Oehler, Carmela Ricciardelli

**Affiliations:** ^1^ Molecular Immunology, Robinson Research Institute University of Adelaide Adelaide SA Australia; ^2^ Reproductive Cancer Research Group, Discipline of Obstetrics and Gynaecology, Adelaide Medical School, Robinson Research Institute University of Adelaide Adelaide SA Australia; ^3^ Chemokine Biology Laboratory, Department of Molecular and Biomedical Science, School of Biological Sciences The University of Adelaide Adelaide SA Australia; ^4^ Carina Biotech, Level 2 Innovation & Collaboration Centre Adelaide SA Australia; ^5^ Department of Gynaecological Oncology Royal Adelaide Hospital Adelaide SA Australia

**Keywords:** 3D‐spheroid, CAR‐T cells, explant assays, immunotherapy, *in vivo*, ovarian cancer

## Abstract

**Objectives:**

Recent studies have identified expression of the non‐functional P2X7 (nfP2X7) receptor on various malignant cells including ovarian cancer, but not on normal cells, which makes it a promising tumour‐associated antigen candidate for chimeric antigen receptor (CAR)‐T‐cell immunotherapies. In this study, we assessed the cytotoxic effects of nfP2X7‐CAR‐T cells on ovarian cancer using *in vitro* and *in vivo* models.

**Methods:**

We evaluated the effects of nfP2X7‐CAR‐T cells on ovarian cancer cell lines (SKOV‐3, OVCAR3, OVCAR5), normal peritoneal cells (LP‐9) and primary serous ovarian cancer cells derived from patient ascites *in vitro* using monolayer and 3D spheroid assays. We also evaluated the effects of nfP2X7‐CAR‐T cells on patient‐derived tissue explants, which recapitulate an intact tumour microenvironment. In addition, we investigated the effect of nfP2X7‐CAR‐T cells *in vivo* using the OVCAR‐3 xenograft model in NOD‐scid IL2Rγnull (NSG) mice.

**Results:**

Our study found that nfP2X7‐CAR‐T cells were cytotoxic and significantly inhibited survival of OVCAR3, OVCAR5 and primary serous ovarian cancer cells compared with un‐transduced CD3^+^ T cells *in vitro*. However, no significant effects of nfP2X7‐CAR‐T cells were observed for SKOV3 or normal peritoneal cells (LP‐9) cells with low P2X7 receptor expression. Treatment with nfP2X7‐CAR‐T cells increased apoptosis compared with un‐transduced T cells in patient‐derived explants and correlated with CD3 positivity. Treatment with nfP2X7‐CAR‐T cells significantly reduced OVCAR3 tumour burden in mice compared with un‐transduced CD3 cells for 7–8 weeks.

**Conclusion:**

This study demonstrates that nfP2X7‐CAR‐T cells have great potential to be developed as a novel immunotherapy for ovarian cancer.

## Graphical abstract

In this study, we have shown that CAR‐T cells targeting the nfP2X7 receptor exhibited anti‐tumour activity against ovarian cancer cell lines and primary serous ovarian cancer cells *in vitro* using monolayer and 3D‐spheroid culture assays. nfP2X7 CAR‐T cells were also effective at inducing apoptosis in patient‐derived explant assays with an intact tumour microenvironment and exhibited anti‐tumour activity *in vivo* using an ovarian cancer xenograft mouse model.

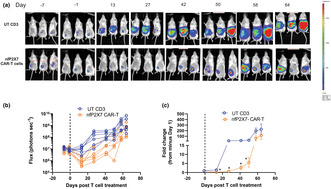

## Introduction

Ovarian cancer is the eighth most common cause of cancer‐related death and second leading cause of cancer‐related mortality among gynaecological cancers in the Western world.^1^ Because of a lack of diagnostic markers and the silent nature of this cancer, most women are diagnosed with advanced disease, which has a 5‐year survival rate of only ~30%.^2^ Despite most patients exhibiting a good initial response to cytoreductive surgery together with platinum and taxane‐based chemotherapy, ~80% of women will relapse and ultimately develop chemotherapy‐refractory disease.^3^ In addition, the 5‐year survival rate for ovarian cancer has not improved over several decades. Recent advances include treatment with poly (ADP‐ribose) polymerase (PARP) and angiogenesis inhibitors; however, resistance to these therapies also develops. Therefore, new and better therapies against ovarian cancer to improve patient outcome are urgently required. T‐cell modulation and the process of redirecting patient T cells to target specific antigens on the surface of tumour cells have been identified as a novel immunotherapy for cancer.^4^, ^5^, ^6^

Advancements in T‐cell modulation to express a chimeric antigen receptor (CAR) that recognises tumour‐associated antigens (TAAs) and enhance cytotoxicity independently of a major histocompatibility complex (MHC), have demonstrated promising responses to manage haematological cancers such as leukaemia and lymphoma.^7^, ^8^, ^9^ Impressive clinical results of CAR‐T‐cell therapy for haematological CD19^+^ B‐cell malignancies have resulted in the FDA approval of several CAR‐T cell therapies, including Yescarta™ (Axicabtagene ciloleucel) and Kymriah™ (Tisagenlecleucel).^10^, ^11^, ^12^ There have been increasing efforts to reproduce these effects in solid tumours, including ovarian cancer.^13^ However, there are many challenges to overcome in order to successfully translate CAR‐T cell therapy to solid cancers.^13^ To ensure successful elimination of malignancies, finding an ideal TAA to target using CAR‐T cells is vital.^14^ An ideal TAA for a CAR‐T‐cell target would be an antigen that is highly expressed on the surface of tumour cells but with little or no expression on normal cells.^15^

We have identified a non‐functional variant of the P2X purinoceptor 7 (P2X7) receptor, nfP2X7, as a potential TAA that could be targeted with CAR‐T cells. The P2X7 receptor belongs to a family of receptors consisting of seven subtypes (P2X1 – P2X7), which are ATP‐ gated cation channels.^16^, ^17^ Upon binding to ATP, activation of P2X7 receptor leads to the formation of pores that allow for the selective influx of calcium (Ca^2+^) into the cell.^16^ In instances of high concentration and prolonged ATP exposure, the P2X7 receptor forms large pores on the cell membrane, which leads to the non‐specific influx of Ca^2+^ and larger molecules of < 900 Da. This results in membrane depolarisation and cell death.^16^ In theory, the high concentration of ATP in the tumour microenvironment (TME) should promote the large pore formation of the P2X7 receptor on cancerous cells and facilitate cell death. Instead, the opposite effects have been observed and high P2X7 receptor expression is actually associated with cell proliferation, survival and metastasis in many solid tumours.^16^ This finding has been attributed to the presence of nfP2X7,^16^, ^18^ which is highly expressed on the surface of many malignant cells, including ovarian cancer cells^16^, ^18^ and is essential for cancer cell survival.^16^ nfP2X7 is thought to be derived from functional P2X7, where the receptor undergoes a conformational change, which expresses a unique epitope (E200) on the cell surface.^16^, ^18^ Furthermore, point mutations, SNPs, splice variants and post‐translational modifications of P2X7 which impair pore formation have also been described.^16^, ^19^ Exposure to high ATP concentration in the TME promotes the expression of nfP2X7 receptor, which display an abnormal trimer formation that prevents the establishment of enlarged pores, therefore allowing the cell to continue to proliferate in high ATP environments.^16^ To date limited studies have examined the expression of nfP2X7 receptor in cancer. nfP2X7 receptor expression was assessed in 70 normal tissues and 290 patient‐derived xenografts including 7 ovarian cancer xenografts.^16^ Up to 40% of all tumours samples showed membrane staining for nfP2X7 receptor^16^ but nfP2X7 receptor was detected in less than 10% of normal tissues.^16^

In this study, we used a second‐generation CAR construct to target the nfP2X7 receptor, using an affinity matured peptide‐binding domain that binds to the nfP2X7 protein. Our previous work showed that CAR‐T cells targeting nfP2X7 receptor (nfP2X7‐M CAR‐T cells) were highly cytotoxic *in vitro* against 12 different solid cancer types, including ovarian cancer.^20^ In addition, significant tumour inhibition was observed against both breast and prostate cancer xenograft mouse models *in vivo*.^20^ In this study, we show efficacy of the nfP2X7‐targeting CAR‐T cells against ovarian cancer cells in monolayer culture, 3D spheroid culture and in patient‐derived explant assays.^17^, ^21^ Furthermore, nfP2X7‐targeting CAR‐T cells significantly reduced tumour burden in an ovarian cancer (OVCAR3) xenograft mouse model *in vivo*.

## Results

### nfP2X7‐CAR‐T cells are cytotoxic against ovarian cancer cells in monolayer culture

The nfP2X7 peptide‐binding domain^16^, ^19^, ^22^ was cloned into a well characterised second‐generation CAR lentiviral backbone encoding a IgG4 hinge/linker and intracellular domains from 41BB and CD3 zeta, connected by a T2A self‐cleaving peptide to a truncated EGFR (EGFRt) reporter as described previously^20^ (Supplementary figure 1). Previous work confirmed that the nfP2X7‐CAR‐T cells act specifically via the P2X7 receptor gene locus and possess on‐target specificity and limited off target cytotoxicity.^20^ Deletion of the *P2X7R* gene in prostate cancer cell line (PC3), significantly reduced the CAR‐T cell‐mediated cytotoxicity, when compared with the wild‐type PC3 cells.^20^ Significant cytotoxicity was observed against cell lines that express nfP2X7 receptor but little cytotoxicity against cell lines that express P2X7 receptor only, or no expression of both nfP2X7 and P2X7 receptor.^20^ There was no *in vitro* cytotoxicity against normal healthy peripheral blood mononuclear cells^20^ that express only wild‐type P2X7 receptor.^17^, ^21^

**Figure 1:**
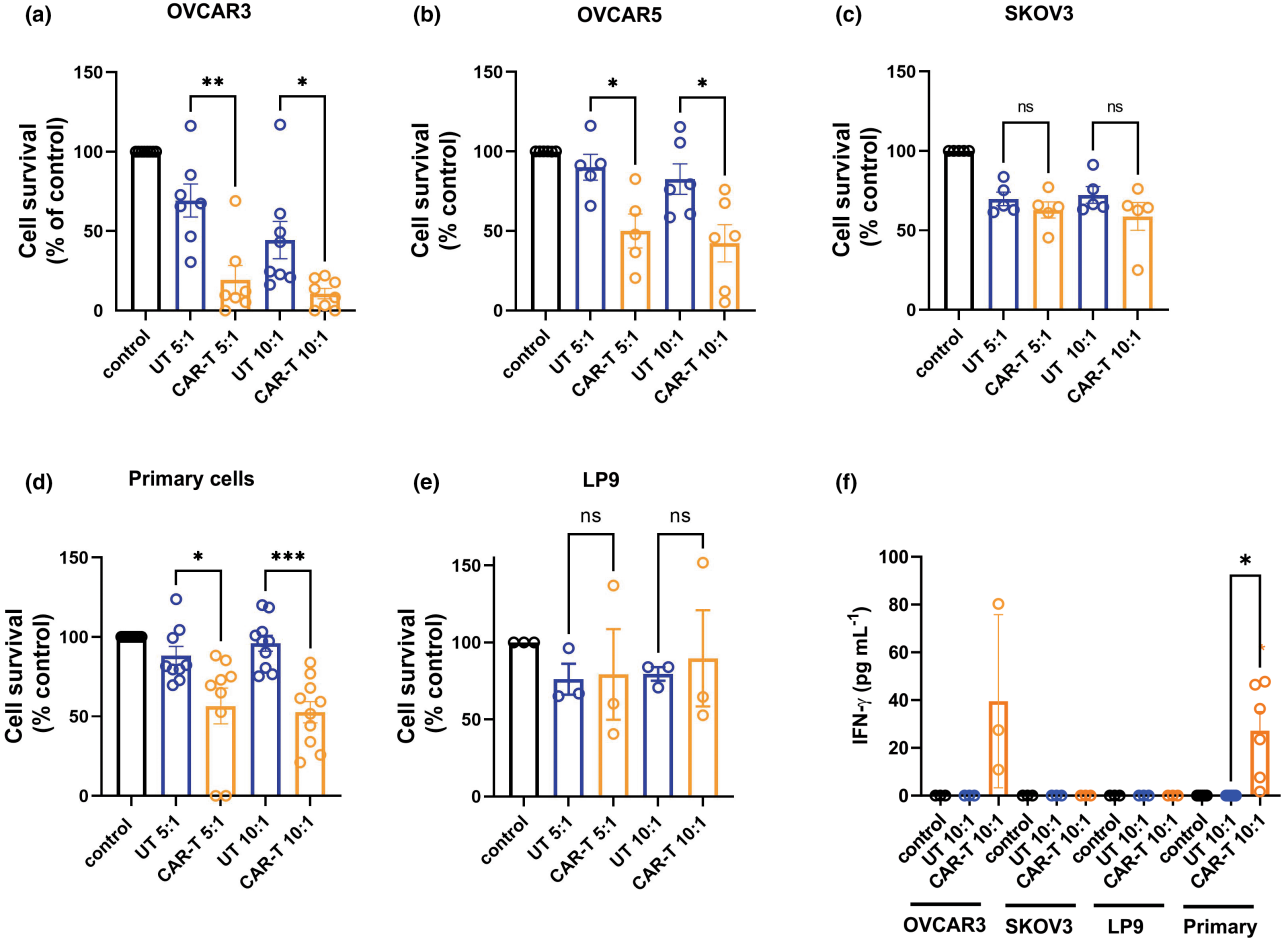
Effect of nfP2X7‐CAR‐T cell treatment on ovarian cancer cell survival in monolayer culture (MTT assay). Survival of **(a)** OVCAR3, **(b)** OVCAR5, **(c)** SKOV3, **(d)** primary human ovarian cancer, **(e)** LP9 normal human mesothelial cells after treatment with control media, nfP2X7 targeting CD3 chimeric antigen receptor T (CAR‐T) cells or untransduced (UT) CD3 cells at a 5:1 or 10:1 effector:target ratio for 48 h and **(f)** INFγ measurements in conditioned media from OVCAR3, SKOV3, LP9 and primary cells following treatment with control media, UT CD3 or nfP2X7 targeting CAR‐T cells at 10:1 effector:target ratio. Data in a‐e represent cell survival as a percentage of the control media only. Data in **(a)** are pooled from 9 independent experiments using 6 different batches of CD3 cells. Data in **(b)** are pooled from 6 independent experiments using 6 different batches of CD3 cells. Data in **(c)** are pooled from 5 independent experiments using 3 batches of CD3 cells. Data in **(d)** are pooled from 7 independent experiments using 6 different batches of CD3 cells and 10 different primary ovarian cancer cell samples. Data in **(e)** are pooled from 3 independent experiments using 2 batches of CD3 cells. Data in **(f)** are pooled from 3 batches of matched UT CD3 or CAR‐T cells for OVCAR3, SKOV3 and LP9 cells and one batch of UT CD3 or CAR T cells for the primary cells (*n* = 6). All data are represented as mean ± SEM. One‐way ANOVA (Tukey's multiple comparisons test, *P < 0.05, **P < 0.01, ***P < 0.001, ns, not significant).

Initially, the cytotoxic potential of the nfP2X7‐targeting CAR‐T cells against ovarian cancer cells were tested using monolayer assays *in vitro*. For this assay ovarian cancer cells were plated in a monolayer, 24 h later nfP2X7 targeting CAR‐T cells or UT (un‐transduced) cells were added at different effector: target (E:T) ratios, then co‐cultured for further 48 h, and the percentage of viable OVCAR3 cells remaining were determined using the MTT assay. A significant decrease in OVCAR3 cell survival was observed at both the 5:1 and 10:1 effector: target (E:T) cell ratios when compared with donor‐matched UT CD3 cells (Figure 1a). Similarly, at both 5:1 and 10:1 E:T ratios, nfP2X7‐targeting CAR‐T cells significantly reduced the survival of OVCAR5 cells when compared with UT CD3 cells (Figure 1b). These findings were consistent with results from a luciferase‐based *in vitro* cytotoxicity assay for both OVCAR3 and OVCAR5 (Supplementary figure 2) and in our previous report.^20^ In contrast, no significant change in the survival of SKOV3 cells were observed after co‐culture with nfP2X7‐targeting CAR‐T cells, when compared with UT CD3 cells (Figure 1c). In addition, nfP2X7‐targeting CAR‐T cells were tested against primary ovarian cancer cells isolated from patient ascites (Supplementary table 1). When tested against primary human ovarian cancer cells (from 10 patients), nfP2X7‐targeting CAR‐T cells were cytotoxic resulting in a significant decrease in primary cancer cell survival at both 5:1 and 10:1 E:T ratios when compared with the UT CD3 cells (Figure 1d, *P*‐value = 0.0162 and *P*‐value = 0.0002 for 5:1 and 10:1, respectively, Tukey's multiple comparisons test, one‐way ANOVA). However, nfP2X7‐CAR‐T cells showed no significant cytotoxic effects against normal human mesothelial cells (LP9) when compared with UT CD3 cells indicating these cells are not a target for anti‐nfP2X7 cytotoxicity (Figure 1e). In addition to *in vitro* cytolysis, there was increased production of the cytokine IFNγ in the supernatants collected from OVCAR3 and primary ovarian cancer cells following 48 h co‐culture with nfP2X7‐CAR‐T cells, but no IFNγ was detected in supernatants from cells treated with control media or co‐cultured with UT CD3 cells (Figure 1e). Consistent with the cytotoxicity assays, no IFNγ was detected in the supernatants collected from SKOV3 or LP‐9 following 48 h co‐culture with nfP2X7‐CAR‐T cells (Figure 1e).

Because of the lack of a commercial antibody for detecting nfP2X7 receptor expression, it was not possible to ascertain the nfP2X7 expression on ovarian cancer cells. We investigated whether cytotoxic activity of the nfP2X7‐CAR‐T cells against ovarian cancer cells in the monolayer assays was related to P2X7 receptor expression. P2X7 receptor expression was assessed in ovarian cancer cells: OVCAR3, OVCAR5, and SKOV3 and the breast cancer cell line MDA‐MB‐231 used as positive control^20^ with an anti‐P2X7 antibody using immunofluorescence. P2X7 receptor expression was observed in OVCAR3, OVCAR5, MDA‐MB231 cancer cell lines and primary ovarian cancer cells (Supplementary figure 3). In contrast, lower P2X7 receptor expression was observed in SKOV3 and normal mesothelial cells (LP9) (Supplementary figure 3).

### nfP2X7‐CAR‐T cells were cytotoxic against ovarian cancer in 3D spheroid culture *in vitro*

Development of malignant ascites that contain multicellular spheroids is common in ovarian cancer patients both at diagnosis but more commonly following relapse.^23^ nfP2X7‐targeting CAR‐T cells were tested against ovarian cancer cells in 3D spheroid cultures, which are more closely represented in ovarian cancer in patients. For this assay, ovarian cancer cells were plated on poly‐HEMA coated 24‐well plates in respective growth media, 24 h later nfP2X7‐CAR‐T cells or UT cells were added to each well, and spheroid formation was observed for 6 days.

To investigate whether there is an advantage of using a 1:1 mix of CD4:CD8 nfP2X7‐CAR‐T cells compared with an unfractionated CD3 nfP2X7‐CAR‐T cell population, each type was added to OVCAR3 spheroid cultures. There was no noticeable advantage of using the 1:1 CD4/CD8 nfP2X7‐CAR‐T cells over the mixed CD3 nfP2X7‐CAR‐T population (Supplementary figure 4a). Both the 1:1 mix of CD4/CD8 nfP2X7‐CAR‐T cells and CD3 nfP2X7‐CAR‐T cells were able to similarly decrease spheroid size of OVCAR3 cells compared with UT CD4/CD8 or UT CD3 cells (Supplementary figure 4a). Therefore, all subsequent spheroid assays were performed with unfractionated CD3 T cells.

**Figure 2:**
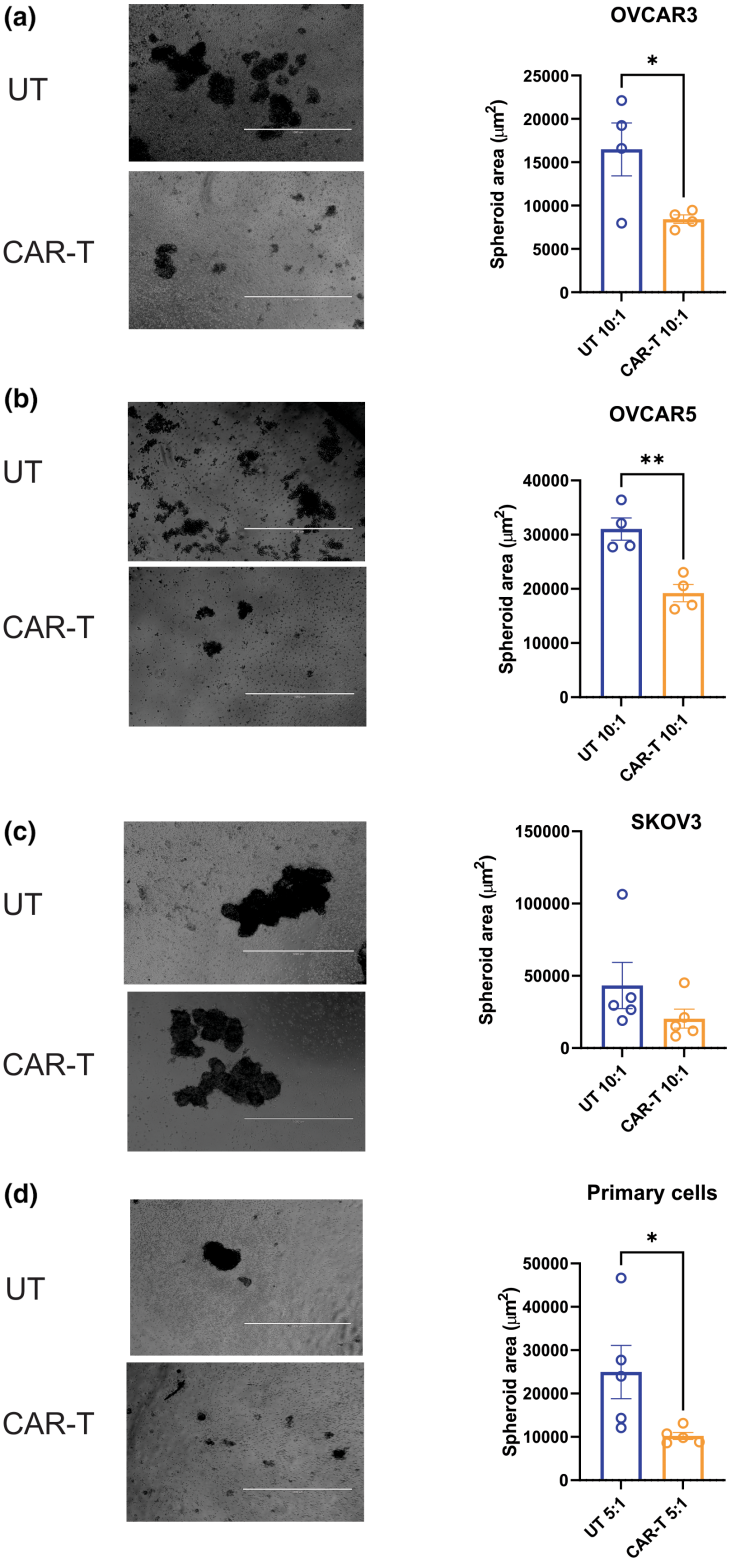
Effect of nfP2X7‐CAR‐T cells on ovarian cancer spheroid culture. Representative images of spheroids (left panels) captured at 72 h post‐treatment and spheroid area quantitation using Image J (right panels). **(a)** Comparison of OVCAR3 cells treated with either nfP2X7‐CAR‐T cells or un‐transduced (UT) CD3 cells. Significant decrease in spheroid area was observed at 10:1 (E:T) ratio. OVCAR3 data represents mean ± SEM of 4 replicates from 2 independent batches of CD3 cells. **(b)** Treatment of OVCAR5 cells with nfP2X7‐CAR‐T cells resulted in a significant decrease in spheroid area compared with UT CD3 cells at 10:1 ratio. Data represent mean ± SEM of 4 replicates from 2 independent batches of CD3 cells. **(c)** SKOV3 cell line did not respond to treatment with nfP2X7‐CAR‐T cells in the 3D spheroid culture. Data represent mean ± SEM of 5 replicates from 3 independent batches of CD3 cells. *P < 0.05, unpaired Student *t*‐test. **(d)** nfP2X7‐CAR‐T cells treated primary cells (5:1) had reduced spheroid size at 72 h of treatment compared with UT CD3 cells. *P < 0.05; **P < 0.01, unpaired Student's *t*‐test. Data were collected from 5 different primary cell cultures using 3 independent batches of CD3 cells. Scale bar = 1000 μm.

Consistent with previous results from the MTT assay, treatment with nfP2X7‐CAR‐T cells resulted in a significant decrease in OVCAR3 spheroid area at 10:1 E:T ratio compared with UT CD3 cells (Figure 2a, *P‐*value = 0.045, Student's *t‐*test). Similarly, OVCAR5 spheroids showed a significant decrease in spheroid area after treatment with nfP2X7‐CAR‐T cells at 10:1 E:T ratio (Figure 2b, *P*‐value = 0.0039) compared with UT CD3 cells. No significant difference was observed in SKOV3 spheroid area after treatment with nfP2X7‐CAR‐T cells at 10:1 E:T ratio (Figure 2c). Treatment with nfP2X7 CAR‐T cells significantly reduced primary ovarian cancer spheroids size from five patients at a 5:1 ratio (Figure 2d, *P‐*value = 0.015, Student's *t‐*test).

### nfP2X7‐CAR‐T cells were cytotoxic against ovarian cancer cells in patient‐derived explant assays

The TME plays a major role in tumour survival and resistance to therapies, by a range of processes, including limiting CAR‐T cell trafficking and tumour inhibition. Patient‐derived cancer tissues, which retain the architecture and some cellular components of the TME closely represent the actual TME *in vivo*.^24^ Therefore, nfP2X7‐CAR‐T cells were tested against ovarian cancer patient‐derived explants (PDE) to assess their ability to specifically home and target ovarian cancer cells in an intact microenvironment.

Briefly, cryopreserved ovarian cancer tissues (Supplementary table 1) were thawed, dissected into 1‐mm^3^ pieces and explanted onto gelatine dental sponges (Spongostan, Johnson & Johnson) in 24‐well plates as described previously.^24^ Sponges were immersed in culture media supplemented with cytokines (IL‐2, IL‐7, and IL‐15). Explants were then treated with PBS, carboplatin, UT T cells or nfP2X7‐CAR‐T cells and incubated in a humidified atmosphere at 37°C containing 5% CO_2_ for 48 h, then processed for histology and immunohistochemistry.

Initially, PDE assays were performed using a 1:1 mix of CD4:CD8 or unfractionated CD3 CAR‐T cells. There were no differences in apoptosis (assessed by cleaved caspase 3 positivity) in the four different ovarian cancer tissues treated with either CD3 or a 1:1 mix of CD4:CD8 nfP2X7 CAR‐T cells (Supplementary figure 4b–e). Therefore, additional experiments were carried out using a 1:1 mix of CD4/CD8 T cells.

**Figure 3:**
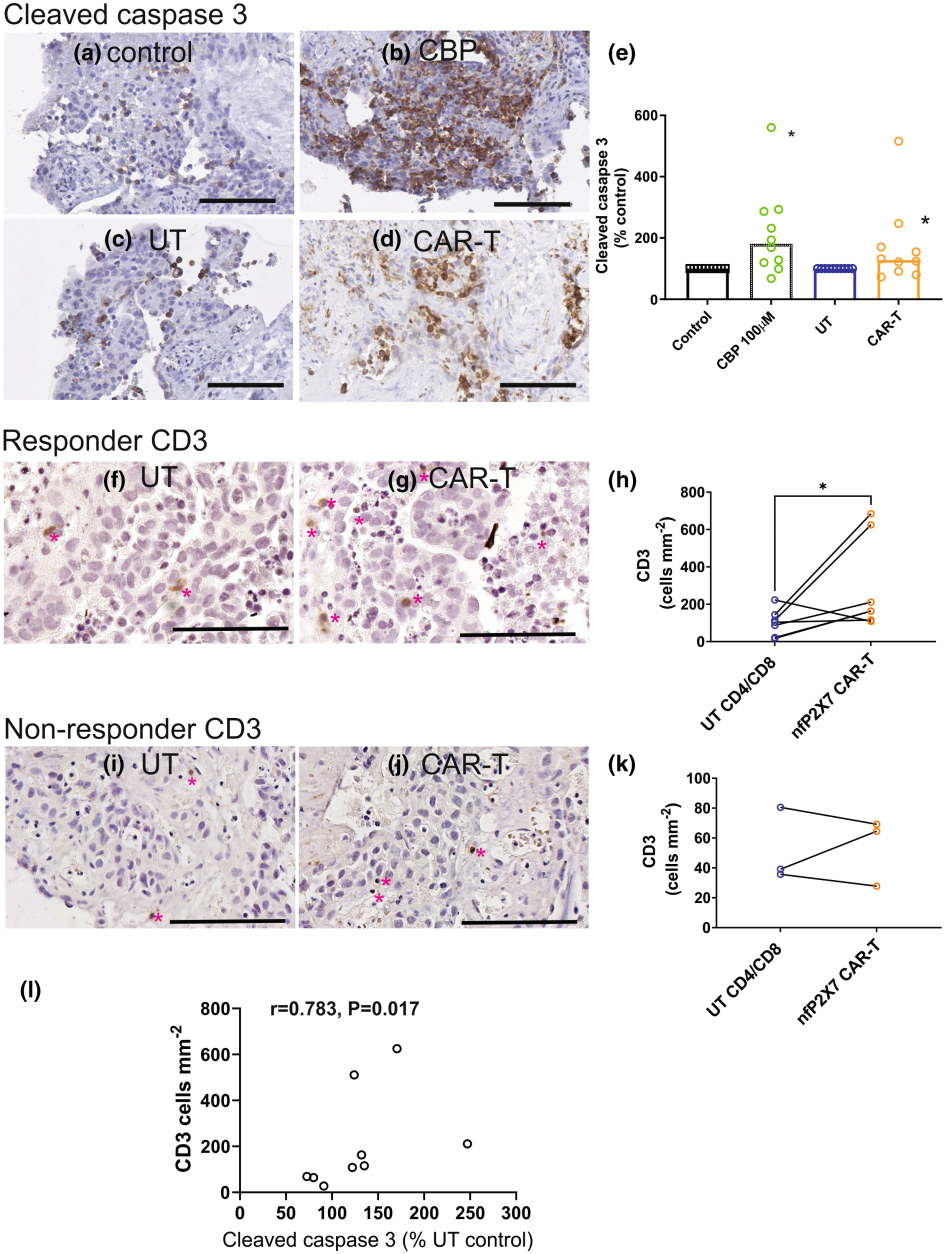
Effect of nfP2X7‐CAR‐T cells in patient‐derived explant assays. Representative images of cleaved caspase 3 immunostaining in patient‐derived ovarian cancer explants (patient 5) following treatment with PBS **(a)**, carboplatin (CBP, 100 μm **(b)**, un‐transduced (UT) T cells, (CD4:CD8 1:1) **(c)**, nfP2X7‐CAR‐T (CD4:CD8 1:1) **(d)** for 48 h. **(e)** Cleaved caspase 3 quantitation. Data are expressed as % of PBS or UT CD3 controls (*n* = 10, patients 4–13), The bar graphs show the median values. *, significantly increased compared to PBS control and UT T cell treatment, Wilcoxon signed‐rank test (*P* < 0.05). CD3 immunostaining in explant tissue that responded to nfP2X7‐CAR‐T treatment (patient 11); treatment with un‐transduced (UT, CD4:CD8 1:1 cells) **(f)** and nfP2X7‐CAR‐T (CD4:CD8 1:1) **(g)** for 48 h. CD3 quantitation using quPath analysis in explant tissues that responded to nfP2X7‐CAR‐T treatment **(h)**, *n* = 7). Data are expressed as % of UT CD3 control. *P = 0.046, Wilcoxon signed‐rank test. CD3 immunostaining in ovarian cancer explant tissue that did not respond to treatment with nfP2X7‐CAR‐T (patient 10) treatment with UT (CD4:CD8 1:1 cells) **(i)** and nfP2X7‐CAR‐T (CD4:CD8 1:1) **(j)** for 48 h. CD3 quantitation using quPath analysis in explant tissues that did not respond to nfP2X7‐CAR‐T treatment **(k)**, *n* = 3). Data are expressed as % of UT CD3 control. **(l)** Correlation between CD3 positivity (cells mm^−2^) and response to nfP2X7‐CAR‐T treatment measured by cleaved caspase 3 positivity (% of UT control), Spearman correlation test, *r* = 0.783, *P* = 0.0172. **(a–d)** scale bar = 50 μm, **(f–j)** scale bar = 100 μm. **(f–j)** pink asterisks indicate CD3 positive cells.

Cleaved caspase 3 positivity was significantly increased with carboplatin, a chemotherapy drug used in the first line treatment for ovarian cancer compared with the PBS control (Figure 3a–e, *P* = 0.0098, Wilcoxon paired test). Cleaved caspase 3 positivity was significantly increased following treatment with nfP2X7‐CAR‐T cells (1:1 ratio CD4:CD8, 2 × 10^6^ cells mL^−1^ compared with UT CD4:CD8 cells) (Figure 3e, *P*‐value = 0.0371, Wilcoxon paired test). Using an arbitrary cut‐off point set to 20% of control, responses to carboplatin and nfP2X7‐CAR‐T were observed in 8/10 (80%) and 7/10 (70%) of patient tissues, respectively (Figure 3e). One patient tissue that did not respond to carboplatin responded to treatment with CD4 CD8 nfP2X7‐CAR‐T cells. P2X7 receptor was expressed in all ovarian cancer tissues included in the explant assays (Supplementary figure 5a); however, no relationship between P2X7 receptor expression and response to nfP2X7‐CAR‐T cell treatment was apparent (Supplementary figure 5b).

CD3 staining of explant tissues were conducted after treatment with UT T cells or nfP2X7‐CAR‐T cells, to confirm T‐cell trafficking into the tumour tissue and also to investigate whether there is a difference between the UT and nfP2X7‐CAR‐T cell infiltration. Although there was some variability in CD3 levels between the explant assays after treatment with UT T cells or P2X7‐CAR‐T cells, we confirmed that CD3 positivity was significantly increased in explant tissues treated with nfP2X7 CAR‐T cells that responded to treatment with nfP2X7 CAR‐T cells (Figure 3f–h, *P*‐value = 0.046) but not in explant tissues that did not respond (Figure 3j and k). A significant positive correlation was observed between CD3 positivity and response to nfP2X7 CAR‐T cells measured by cleaved caspase 3 positivity (Figure 3l, Spearman correlation *r* = 0.783, *P*‐value = 0.0172).

### nfP2X7‐CAR‐T cells inhibited tumour growth in OVCAR3‐bearing NSG mice

Next, we investigated the *in vivo* anti‐tumour efficacy of nfP2X7 CAR‐T cell therapy in an ovarian cancer xenograft mouse model. NSG mice were inoculated with OVCAR3‐luc cells that stably expressed luciferase via i.p. injection and on day 21 post‐tumour injection, either UT or nfP2X7‐CAR‐T CD3 cells (1 × 10^7^ cells/mouse, Batch 21) were delivered by i.p. injection. Characterisation of batch 21 cells prior to injection showed they were 63% CAR positive and consisted of 48.2% CD4 and 50.5% CD8 cells (Supplementary figure 6). These contained a significant proportion of cells with a central memory phenotype (50% and 27% for CD4 and CD8 cells, respectively) (Supplementary figure 6). We also looked at surface expression of T‐cell inhibitory markers PD1 and CTLA4 in CD4 and CD8 cells. We saw high PD1 expression in both CD4 and CD8 T cells from batch 21, and PD1 expression was higher in the nfP2X7‐CAR‐T cells than UT cells (Supplementary figure 7 and Supplementary table 2). CTLA4 is another receptor expressed by T cells which binds to B7 expressed on antigen presenting cells and cancerous cells, resulting in T‐cell inhibition.^25^ We saw low levels of CTLA4 in both the CD4 and CD8 population (Supplementary figure 7).

**Figure 4:**
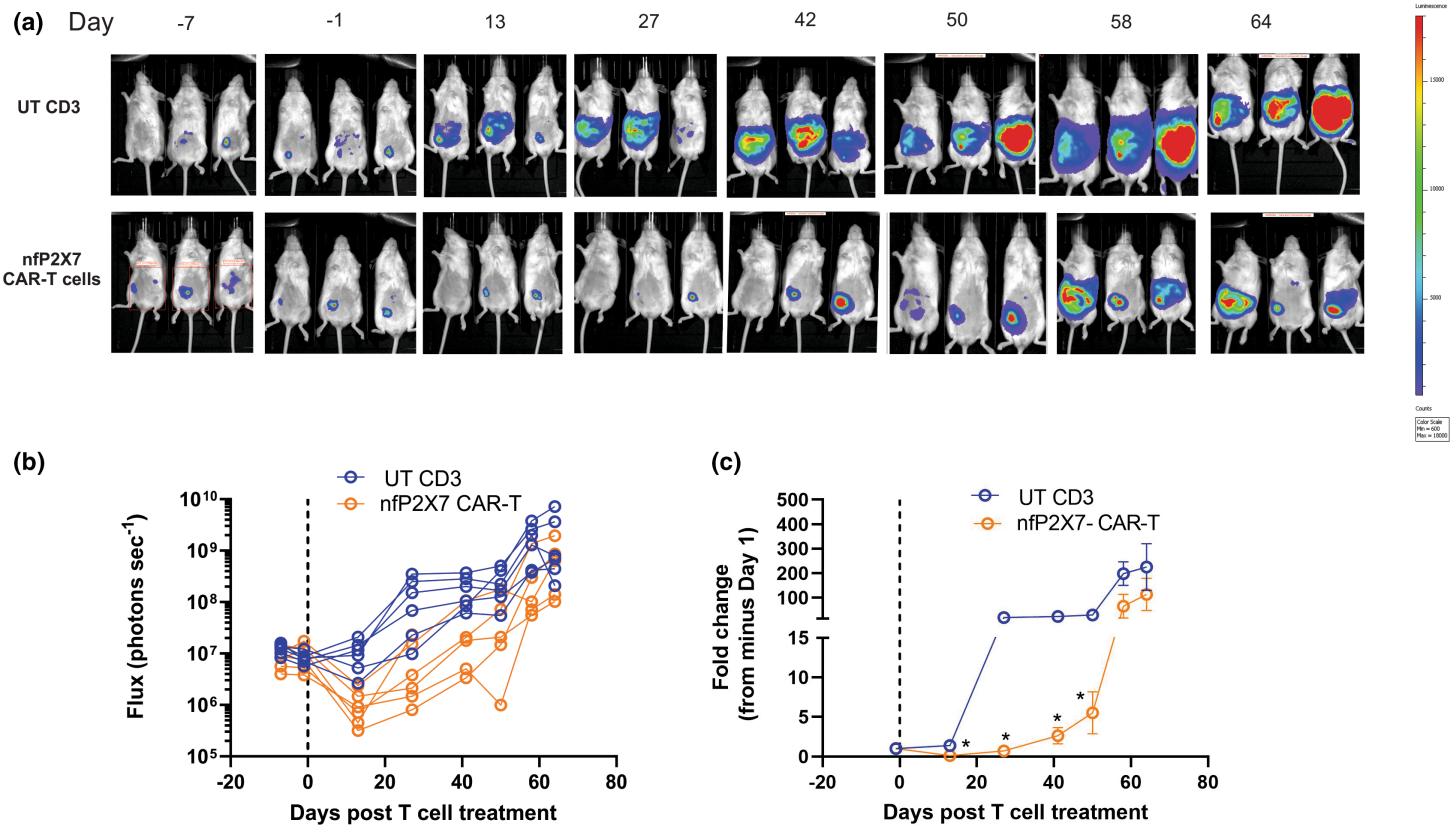
Effects of intraperitoneal (i.p.) delivery of nfP2X7 CAR‐T cells on ovarian cancer metastasis in OVCAR3‐luc xenografts (Experiment 1). **(a)** Representative bioluminescence flux imaging from OVCAR3‐luc tumour‐bearing NSG mice injected with 5 × 10^6^ OVCAR3 cells followed by the administration (i.p) with either 1 × 10^7^ cells un‐transduced (UT) CD3 cells or nfP2X7‐CAR‐T cells on day 21 post‐tumour injection (Day 0). IVIS imaging from −day 7 to day 64 post‐treatment. **(b)** Quantification of flux (each mouse) from OVCAR3‐luc bearing mice treated i.p. with CD3 UT cells (*n* = 6) or nfP2X7‐CAR‐T cells (*n* = 6), data from one independent experiment. Data are flux (photons s^−1^) for each mouse. **(c)** Average fold change in flux (from minus day 1) of OVCAR3‐luc bearing mice. i.p delivery of nfP2X7 CD3 CAR‐T cells at day 0 significantly reduced tumour burden in OVCAR3‐luc tumour‐bearing mice, compared with UT CD3 cells, at day 13, 27, 41 and 50. Error bars mean ± SEM, *P < 0.05, Student's‐*t*‐test at each time point. The dotted line indicates time of CD3 cell administration.

Representative images of mice at minus D7, minus D1, and following treatment with UT or nfP2X7‐CAR‐T CD3 cells (days 13–64) are shown in Figure 4a. At D13, a significantly reduced tumour burden was observed in OVCAR3‐luc mice treated with nfP2X7‐CAR‐T cells compared with UT CD3 cells. The effect remained significant at D27, D42, and D50 but was not significant at D58 and D64 post CAR‐T‐cell treatment (Figure 4b). In summary, the nfP2X7‐CAR‐T cell treatment was able to significantly inhibit the OVCAR‐3 tumour growth up to 50 days, but it was not able to completely eradicate the tumours. In an independent *in vivo* experiment, nfP2X7‐CAR‐T and UT CD3 cells (1 × 10^7^ cells/mouse, Batch 30) were injected on day 7 post‐tumour injection. Similar to the first *in vivo* experiment, this batch of nfP2X7‐CAR‐T cells significantly inhibited OVCAR3 tumour growth for up to 56 days (Supplementary figure 8, Experiment 2).

### Characterisation of OVCAR3 xenograft tumours

**Figure 5:**
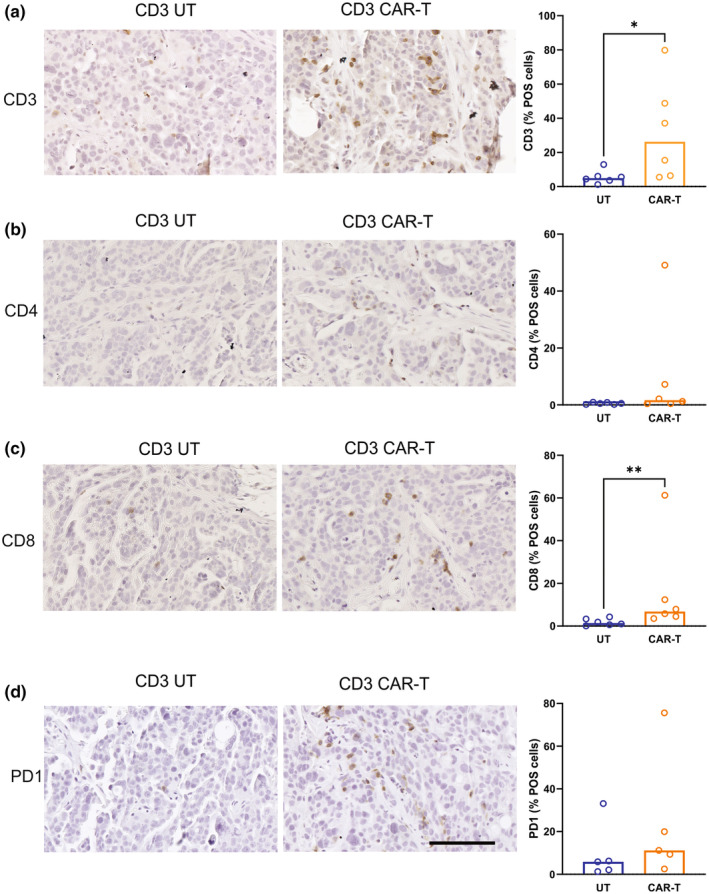
Characterisation of OVCAR3 xenograft tumours. Representative images and quantitation of CD3 **(a)**, CD4 **(b)**, CD8 **(c)** and PD1 **(d)** immunostaining in OVCAR3 xenograft tumours from mice treated with UT CD3 T cells or nfP2X7‐CAR‐T cells. OVCAR3 tumours were collected between 106 and 139 days post T‐cell treatment. All images are the same magnification. Scale bar = 100 μm. Data are the % positive CD3, CD4, CD8, or PD1 cells in tumour area. The bar represents the median value of 5 or 6 tumours/group. *P < 0.05, **P < 0.001. Mann–Whitney *U*‐test.

*Ex vivo* analysis of OVCAR3 tumours at endpoint (up to 139 days post T‐cell injection) showed significantly higher frequencies of human CD3^+^ and CD8^+^ T cells, in mice administered with nfP2X7 CAR‐T cells than in those administered with UT cells (Figure 5a and c). Although the presence of CD4^+^ and PD1^+^ cells were confirmed, there was no significant difference in the frequency of CD4^+^ (Figure 5b) and PD1^+^ (Figure 5d) cells in the OVCAR3 tumours obtained from mice that received either UT or nfP2X7‐CAR‐T cells.

## Discussion

CAR‐T cell immunotherapy has shown great promise in the treatment of haematological cancers. However, there has been limited success in treating solid tumours due to challenges, such as the difficulty in identifying TAA targets, overcoming the suppressive TME and enabling T‐cell trafficking and infiltration into the tumour for effective inhibition of solid tumours. Most ovarian cancer patients are not diagnosed until the disease has reached an advanced stage resulting in overall poor prognosis. Therefore, effective novel therapies are needed to achieve a long‐term favourable clinical outcome for ovarian cancer patients. Previous small clinical trials with adoptive cell transfer (ACT) of autologous tumour‐infiltrating lymphocytes (TILs) in patients with ovarian cancer were found to be safe but response rates were variable.^5^ For T‐cell‐based cancer therapy to be more successful, a more effective patient‐specific approach to find the most relevant TAAs in an individual's tumour need to be developed and targeted prior to T‐cell selection.^26^ However, not all cancers contain sufficient TILs, and the technical difficulty associated with identifying, isolating, and expanding tumour‐reactive lymphocytes has encouraged the development of engineered T cells.

Antigen specific CAR‐T immunotherapy for ovarian cancer holds great potential.^13^ There has been some success in targeting several TAAs in preclinical ovarian cancer studies including MUC16,^27^ mesothelin,^28^, ^29^, ^30^ follicle stimulating hormone receptor^31^, ^32^ and folate receptor α.^33^ Several clinical trials are currently underway or have been completed with ovarian cancer patients using CAR‐T cells targeting some of these TAAs.^13^, ^34^ Our previous study has shown that CAR‐T cells targeting nfP2X7 receptor have anti‐tumour activity against breast (MDA‐MB231 cell line) and prostate (PC3 cell line) cancer xenograft models *in vivo*.^20^ Results from this this study shows that CAR‐T cells targeting the nfP2X7 receptor exhibited anti‐tumour activity against both ovarian cancer cell lines (OVCAR3 and OVCAR5) and primary serous ovarian cancer cells *in vitro* using monolayer and 3D‐spheroid culture assays. nfP2X7 CAR‐T cells were also effective at inducing apoptosis in patient‐derived explant assays with an intact TME. Furthermore, nfP2X7 CAR‐T cells exhibited anti‐tumour activity in the *in vivo* OVCAR3 xenograft mouse model in NSG mice. These findings indicate that nfP2X7 CAR‐T cells have potential to be used as a novel treatment against ovarian cancer.

A second‐generation CAR construct that expresses the nfP2X7 antigen‐binding domain with a 4‐1BB intracellular costimulatory domain was used in this study, which has been shown to promote T‐cell differentiation into a central memory subset phenotype and increased persistence *in vivo*.^20^, ^35^, ^36^ This CAR construct was based on the same lentiviral backbone as a FDA‐approved CD19 CAR‐T therapy, used to treat haematological malignancies such as leukaemia and lymphoma.^37^ Initial experiments investigated the effectiveness of a 1:1 mixture of CD8^+^ and CD4^+^ CAR‐T cells compared with unsorted CD3 CAR‐T cells, as previous studies suggested a pooled CD4/CD8 CAR‐T therapy is more potent *in vivo* than CD8 alone.^8^ In this study, similar efficacy was observed between the 1:1 mix of CD4:CD8 nfP2X7 CAR‐T cells and unsorted nfP2X7 CD3 T cells in both 3D spheroid and patient‐derived explant assays. Since there was no significant advantage of using a 1:1 mix of CD4:CD8 cells a protocol was developed to facilitate the co‐generation of donor‐matched CD4 and CD8 CAR‐T cells simultaneously by using CD3 T cells. This protocol simplified the CAR‐T cell manufacturing without the need for separate purification and transduction protocols. CD3 CAR‐T cells are more easily produced in the laboratory, required less processing and are more feasible to generate sufficient numbers for clinical studies. However, a limitation to using CD3 T cells was the inability to directly control the ratio of CD4:CD8 populations in the cell culture.

In this study, UT CD3 cells were used as a negative control, which have also been used in our^20^ and other recently published studies.^38^, ^39^, ^40^, ^41^, ^42^ However, UT CD3 cells are not able differentiate any changes in T‐cell activity because of lentiviral transduction. Results from this study and previous work confirm that the CAR‐T effect is nfP2X7 specific, as the nfP2X7‐CAR‐T cells did not react to all cancer type tested against or PBMC cells that only express P2X7 receptors.^20^ Furthermore, we showed that CAR‐T‐cell cytolysis was nfP2X7 specific as cytolysis was observed against prostate cancer cells when nfP2X7 gene was deleted.^20^ Future studies would benefit from inclusion of a truncated endodomain that is signalling defective, an ectodomain control targeting an irrelevant antigen and vector‐alone transduced T cells to account for any changes as a result of transduction.

This study showed significant anti‐tumour activity of nfP2X7 CAR‐T cells *in vitro* against primary ovarian cancer cells in the 3D spheroid cultures. Assessment of CAR‐T cell‐mediated killing of ovarian cancer spheroids is highly relevant as up to one third of ovarian cancer patients at diagnosis, and most ovarian cancer patients at recurrence develop malignant ascites.^23^ Ascites contain multicellular spheroids, which are enriched in cancer stem‐like cells and contribute to ovarian cancer metastasis and chemotherapy resistance.^43^ Therefore, using 3D spheroid cultures of primary ovarian cancer to assess anti‐tumour activity of nfP2X7‐CAR‐T cells is important, as it more closely represents ovarian cancer *in vivo*.

In this study, we did not have the ability to detect and characterise nfP2X7 expression in ovarian cancer cells and tumour tissues, as a nfP2X7 antibody is not commercially available. We assessed expression of P2X7 receptor in ovarian cancer cell lines and tissues by immunocytochemistry and immunohistochemistry using a commercially available polyclonal antibody from Novus Biologicals (NBP2‐19654). This polyclonal antibody was raised to the centre region of the P2X7 receptor and therefore most likely binds to both P2X7 and nfP2X7 receptors and therefore cannot differentiate between the expressions of wild‐type P2X7 versus nfP2X7 receptor. We found abundant expression of P2X7 receptor in ovarian cancer cells OVCAR3 and OVCAR5 but not SKOV3 or normal mesothelial cells (LP9). Our *in vitro* studies demonstrating anti‐tumour activity in both OVCAR3 and OVCAR5 cells but not SKOV3 cell and normal LP‐9 mesothelial cells were consistent with the P2X7 receptor expression by immunocytochemistry. P2X7 receptor was expressed in all ovarian cancer tissues included in the patient‐derived explant assays. However, nfP2X7 CAR‐T cells were not cytotoxic towards some ovarian tissues even though they expressed P2X7. The most likely explanation for this is that these ovarian cancer cells express the wild‐type P2X7 receptor that is not recognised by the nfP2X7‐CAR‐T cells. This view is supported by our findings that CD3 positivity was increased in explant tissues that responded to the nfP2X7 CAR‐T cells and correlated with the response observed with nfP2X7‐CAR‐T‐cell treatment measured by cleaved caspase‐3 positivity. A monoclonal antibody raised against a P2X7 amino acid sequence (200–216), whose conformation is distinct from that of wild‐type P2X7 has been shown to bind specifically to nfP2X7 expressed on the surface of tumour cells.^16^ Future studies using a nfP2X7 specific antibody will be helpful to characterise nfP2X7 expression in different ovarian cancer subtypes and its prognostic significance.

The *in vivo* study reported here showed that nfP2X7‐CAR‐T cells were able to significantly inhibit OVCAR3 tumour growth. However, the anti‐tumour effect of nfP2X7 CAR‐T cells was lost after 50 days post CAR‐T‐cell treatment. At the experimental endpoint, we did detect CD3 T cells in OVCAR3 tumour tissues even after 139 days post‐T‐cell treatment. Further analysis showed that there were more CD3 and CD8 T cells but not CD4 T cells, in OVCAR3 tumours from mice treated with nfP2X7 CAR‐T cells when compared with UT CD3 T cells. This observation suggests that either nfP2X7‐CAR‐T cells infiltrated the OVCAR3 tumour tissue more efficiently when compared with the UT CD3 T cells, or that nfP2X7‐CAR‐T cells expanded within the tumour as a result of binding with the target antigen. However, although more CD3 and CD8 T cells were found within the tumours of mice treated with nfP2X7‐CAR‐T cells, the T cells appeared to have lost the ability to inhibit tumour growth either through T‐cell exhaustion or suppression, loss of target antigen or a combination of both. Phenotype analysis of cells used in this study (batch 21) showed that there was a high percentage of CD4 and CD8 T cells expressing an inhibitory receptor PD1; this may be responsible for early onset of T‐cell exhaustion. The CD3 T cells harvested from OVCAR3 tumours at the endpoint also showed PD1 expression, suggesting T‐cell exhaustion as a possible cause for the inability to completely inhibit OVCAR3 tumour growth in this model. Improvements in CAR‐T‐cell expansion methods to overcome T‐cell exhaustion may also improve the quality and longevity of treatment.^44^ These include different T‐cell activation strategies, different cytokine supplementation, and inhibiting T‐cell differentiation.^44^

In this study, the selected method of T‐cell delivery was by i.p. injection. This method was selected as a previous study by Murad *et al*. (2018)^45^ comparing the efficacy of administering CAR‐T cells targeting tumour‐associated glycoprotein 72 (TAG72) in OV90 xenografts using intravenous and i.p. injections showed that i.p. delivery of CAR‐T cells resulted in overall better anti‐tumour activity in OV90‐bearing NSG mice.^45^ A repeat dose of CAR‐T cells also improved the survival of the OV90 bearing mice, which may be required to maintain anti‐tumour activity in the OVCAR3 using nfP2X7‐CAR‐T cells.^45^ Furthermore, combining nfP2X7 CAR‐T cells with other treatments may also help to maintain the anti‐tumour activity over a longer period. A study by Cherkassky *et al*. (2016)^46^ combined PD1 checkpoint blockade with CAR‐T‐cell therapy and found that this combination enhanced T‐cell function and reduced PD1‐mediated exhaustion.^46^ Another improvement would be to develop dual CAR‐T cells targeting nfP2X7 with another TAA such as MUC16. A recent study found that dual CAR‐T cells targeting both PDL1 and MUC16 exhibited enhanced anti‐tumour activity both *in vitro* and *in vivo* against OVCAR3 cells compared with single CAR‐T cells.^27^

## Conclusions

This study demonstrates that the nfP2X7 receptor has great potential to be a successful TAA for CAR‐T‐cell immunotherapy in ovarian cancer patients. Future studies using patient‐derived xenografts are required to support the development of nfP2X7‐CAR‐T‐cell therapy for patients with ovarian cancer.

## Methods

### Cell culture

Human ovarian cancer cell lines SKOV3 were purchased from American Type Culture Collection (ATCC, VA, USA). OVCAR‐3‐Luc were obtained from Japanese Collection of Research Bioresources Cell Bank (Cellbank Australia, Westmead, NSW, AUS) OVCAR5 cells were obtained from Dr Thomas Hamilton (Fox Chase Cancer Center, PA, USA). OVCAR5, OVCAR3, and SKOV3 cell lines were grown in RPMI 1640 media (Sigma Aldrich, Burlington, MA, USA). Growth media was supplemented with 4 mM L‐glutamine, 10% foetal bovine serum (FBS, Scientifix, Clayton, VIC, Australia) and PSF antibiotics (100 U penicillin G, 100 μg mL^−1^ streptomycin sulfate and 0.25 μg mL^−1^ amphotericin B, Sigma Aldrich). LP‐9 peritoneal cells isolated from human omentum were purchased from Coriell Cell Repository (Camden, USA) and maintained in a 1:1 mixture of M199 medium (Sigma Aldrich) and Ham's F‐12 nutrient medium (Sigma Aldrich) supplemented with 10% FBS, epidermal growth factor (10 ng mL^−1^, Sigma Aldrich), hydrocortisone (0.5 μg mL^−1^, Sigma Aldrich) antibiotics penicillin–streptomycin 1:100 (Sigma Aldrich, cat no.: P4458) and antimycotic (1:500, Sigma Aldrich, cat no.: A5955). All cell lines were determined to be mycoplasma‐free and verified by short tandem repeat (STR) analysis in the last 12 months before use.

The primary culture method has been previously described.^47^ Briefly, tumour cells from ascites were cultured in advanced RPMI‐1640 medium (Life Technologies, Thermo Fisher Scientific, Waltham, MA, USA) supplemented with 2 mmol L^−1^ GlutaMAX (Life Technologies), 10% FBS and antibiotics. Primary ovarian cancer cell lines were stored in liquid nitrogen passage 0–2 and were used in subsequent experiments between passages 1 and 4. Supplementary table 1 summarises the clinical and pathological characteristics of the ovarian cancer patients whose ascites was used to isolate the primary cells. All primary cells showed evidence of epithelial morphology (Supplementary figure 9), which was also confirmed by cytokeratin immunocytochemistry. All cell cultures were maintained at 37°C in an environment of 5% CO_2_.

### P2X7 immunocytochemistry

Cancer cell lines (OVCAR5, OVCAR3, SKOV3, and MDA‐MB231), LP‐9 peritoneal cells and primary ovarian cancer cells were plated (2 × 10^4^ cells/well) in 8‐well tissue culture chamber slides (NunclonTM Lab‐Tek II Chamber slide, RS Glass Slide, Naperville, IL, USA) for 24–48 h. Cells were washed with cold PBS (3×) and fixed with cold 100% methanol (3 min) and cold 100% acetone (1 min), washed with PBS (2 × 5 min), blocked with 5% goat serum and incubated overnight with rabbit polyclonal antibody to P2X7 receptor (1/200, NBP2‐19654, Novus Biologicals, Littleton, CO, USA) followed by goat anti‐rabbit‐Alexa Fluor® 488 (1/200, Molecular Probes, Life Technologies) or goat anti‐rabbit‐Alexa Fluor® 594 (1/200, Molecular Probes, Life Technologies) for 1 h at room temperature and slides mounted with ProLong Gold Antifade Mountant with DAPI (Molecular Probes, Life Technologies). Cells were viewed with an epifluorescence microscope (BX50, Olympus Australia, Parkside, SA, AUS) and imaged using a 40× objective and a Spot RT digital camera (Diagnostic Instruments, Sterling Heights, MI, USA).

### CAR construct design and lentivirus manufacture

The nfP2X7‐binding domain^16^, ^19^, ^22^ was cloned into a second‐generation CAR lentiviral backbone encoding a IgG4 hinge/linker and intracellular domains from 41BB and CD3 zeta, connected by a T2A self‐cleaving peptide to a truncated EGFR (EGFRt) reporter^8^, ^48^, ^49^, ^50^ as described previously.^20^ The nfP2X7 (nfP2X7‐M, IgG4 hinge‐CH3) was used in this study (Supplementary figure 1).^20^ Lentivirus was produced by transfecting 293T cells with the third‐generation self‐inactivating lentiviral plasmid and the packaging plasmids encoding REV, VSV‐G and gag‐pol using established methods.^20^, ^51^, ^52^ Supernatants collected after 24 h and 48 h were concentrated by ultracentrifugation at 20 000 rpm for 2 h at 4°C. The pellet was resuspended in Opti‐MEM reduced serum media (Life Technologies) and stored at −80°C.

### T‐cell transduction and expansion protocol

T cells were isolated from whole blood using RosetteSepTM Human CD4, CD8 or CD3 T‐cell enrichment cocktail (StemCell Technologies, Tullamarine, VIC, AUS) from donors following the manufacturer's protocol. T cells were stimulated with anti‐CD3/CD28 Dynabeads (Gibco, Thermo Fisher), then transduced with lentivirus LV‐nfP2X7 in the presence of polybrene (Sigma Aldrich) at a final concentration of 8 μg mL^−1^ as previously described.^20^ Cells were cultured in complete X‐VIVO™ media (Lonza, Bioscience Australia, Bella Vista, NSW, AUS) supplemented with 2 mM HEPES (Gibco) and 5% human‐serum (cat. #H4522‐100 Sigma Aldrich) or RPMI with 10% FBS and the following cytokines (IL‐2, IL‐7 and IL15) (Lonza or Miltenyi Biotec, Macquarie Park, NSW, Australia). On day 7, transduction efficiency was determined via surface staining with an anti‐EGFR antibody (eBiosciences, Thermo Fisher) and analysis by flow cytometry. Cultures were further expanded by re‐stimulation with peripheral blood mononuclear cells (PBMC), which were irradiated at 40 Grey, and cultured for up to 14 days in a G‐Rex6® well plate (Wilson Wolf, St Paul, MN, USA) with media changes and cytokine additions (every 2–3 days). Cell counts were performed 14–15 days post‐irradiated PBMC stimulation.

Characteristics of the different batches of nfP2X7 CAR‐T cells used in this study are summarised in Supplementary table 2. Transduction efficiency measured by EGFR expression in the different CAR‐T batches varied between 45 and 95% for the CD4^+^ population and between 39 and 100% for the CD8^+^ population (Supplementary table 2). A large variation in PD‐1 expression was observed between the different batches of CAR‐T cells (Supplementary table 2). This varied from 0 to 67.9% in the CD4^+^ population and between 1.0 and 68.0% in the CD8^+^ population (Supplementary table 2).

### BrightGlo luciferase cell survival assay

All batches of CAR‐T cells were tested for their killing ability on the OVCAR3 or OVCAR5 cell line using the BrightGlo Luciferase assay (Promega, Madison, WI, USA). OVCAR3 or OVCAR5 cells that stably expressed luciferase (1 × 10^4^) were seeded (50 μL) into a round bottom 96‐well plate in triplicate for each condition tested. Un‐transduced (UT) T cells or CAR‐T cells were added to the ovarian cancer cells (50 μL) at the following ratios (effector T cells:target cancer cells; E:T) 10:1, 3.1, 1.1. The 96‐well plates were incubated for 16 h at 37°C with 5% CO_2_. On the next day, an equal volume of BrightGlo assay substrate (100 μL) (Promega) was added to the cells of each well before resuspension and incubation for 4 min at room temperature. A volume of 100 μL of the mix was transferred to an opaque plate. The luminescence was measured using a luminometer (GloMax, Promega). The luminescence measured from remaining live target cells was compared with the luminescence from target cells alone to calculate the percentage cytotoxicity of the CAR‐T cells and UT T cells.

### Flow cytometry (FACS)

1–2 × 10^5^ UT T (CD3, CD4 or CD8) cells or nfP2X7‐CAR‐T (CD3, CD4, or CD8) cells were washed in PBS and resuspended in 50 μL Fixable Viability Stain 780 (1:1000) and Human Fc Block (FB, BD Bioscience, San Diego, CA) then incubated for 15 min at room temperature. Cells were washed in 1 × FB (FACS buffer, 1% BSA, 0.04% Na azide in PBS) and stained with antibodies directly conjugated to fluorophore for 30 min at RT. For the subset staining panel: CD3 (clone UCHT1, 1:8 dilution from manufacturer recommended test size), CD4 (BUV496, clone SK3, 1:4), CD8 (BUV395, clone RPA‐T8, 1:8), CCR7 (PE, clone 2‐L1‐A, neat), CD45RA (BB515, clone HI100, 1:6), CD45RO (PeCy7, clone UCHL1, neat), CD62L (BV421, clone DREG‐56, 1:2), and EGFR (eF660, clone me1B3, neat). For exhaustion staining panel: CD3 (BUV737, clone UCHT1, 1:8), CD4 (BUV496, clone SK3, 1:4), CD8 (BUV395, clone RPA‐T8, 1:8), PD‐1 (PECy7, clone EH12.1, neat), CTLA‐4 (BV421, clone BNI3, neat) and CD39 (PE‐CF594, clone TU66, neat) (Becton Dickson Biosciences, *Franklin Lakes, NJ, USA*). Cells were washed once with 1 × FB, washed once in PBS and then fixed in 200 μL 1% paraformaldehyde.

For intracellular cytokine staining, cells were incubated at 37°C, 5% CO_2_ for 4 h in complete IMDM (Gibco) supplemented with 10% FBS (Sigma Aldrich), 1 × penicillin/streptomycin (Gibco), 1× GlutaMAX (Gibco), 54pM β‐mercaptoethanol (Sigma Adrich), 50 ng mL^−1^ phorbol 12‐myristate 13‐acetate (Life Technologies), 1 nM ionomycin (Life Technologies), 1/1500 GolgiStop (BD Biosciences) and 1/1000 GolgiPlug (BD Sciences). After direct staining of surface proteins with fluorophore‐conjugated antibodies, cells were incubated with Cytofix/Cytoperm (BD) for 20 min, washed in Permwash buffer (BD) and stained with an intracellular directly conjugated antibody for 30 min. Cells were washed in PBS 0.04% azide, fixed in PBS 1% paraformaldehyde and stored at 4°C in the dark. Flow cytometry stains were acquired on the BD LSRFortessa X‐20 flow cytometer (Becton Dickson Biosciences) and analysed with FlowJo Software V10.7 (Tree Star).

### 3‐(4,5‐dimethylthiazol‐2‐yl)‐2,5‐diphenyl‐2H‐tetrazolium bromide (MTT) cell survival assay

Ovarian cancer cell lines (OVCAR3, OVCAR5, SKOV3), normal mesothelial cells (LP9) and primary ovarian cancer cells (*n* = 10, Supplementary table 1) were plated at 10 000 cells/well in 96‐well plates in respective growth media. After overnight incubation, cells were treated with control media, UT CD3 cells or nfP2X7‐CAR‐T cells (effector T cells:target cancer cell ratio (E:T) at 5:1 and 10:1) for 48 h. Cell media was removed and thiazolyl blue tetrazolium bromide (MTT) (0.5 mg mL^−1^ in respective media, Sigma Aldrich) was added for 4.5 h and MTT solvent (0.1 N HCl in isopropanol) was added for 10 min before absorbance readings were measured at 595 nm by the Triad series multimode detector plate reader (Dynex technologies, VA, USA). The % of viable cells remaining was calculated from treatment with control media alone.

### IFNγ cytokine release assay

Activation of CAR‐T cells was assessed by measurement of IFNγ release. OVCAR3, SKOV3, LP9 and primary ovarian cancer cells (*n* = 6) were co‐cultured with control media, control CD3 T cells (UT) or nfP2X7‐CAR‐T cells at an E:T at 10:1 for 48 h. The concentration of IFNγ in the supernatant was measured as described previously.^20^

### Spheroid assays

Primary ovarian cancer cells (20 000 cells/well, *n* = 5) and ovarian cancer cell lines (OVCAR3, OVCAR5, and SKOV3 cells, 20 000 cells/well) were plated on poly‐HEMA (30 mg mL^−1^ in 95% ethanol, Sigma Aldrich) coated 24‐well plates in respective growth media. After 24 h, cells were treated with control complete X‐VIVO media with cytokines IL‐2 (50 U mL^−1^), IL‐7 (5 ng mL^−1^) and IL‐15 (0.5 ng mL^−1^) alone or with UT CD3 cells or nfP2X7‐CAR‐T cells (at ratios of 5:1 or 10:1 E:T) resuspended in complete X‐VIVO media. Spheroid formation was observed over 72 h and bright field images were taken using the EVOS® light microscope FL Imaging System (Life Technologies) using a 4× objective zoom. The spheroid area (μm^2^) was determined for each of the treatment groups (*n* = 5 images/well) using Image J 32 software (Image J I.50i, National Institute Health, Bethesda, MD, USA).

### Patient‐derived explant assays

Ovarian cancer tissue samples (*n* = 10) cryopreserved in liquid nitrogen in RPMI containing 15% DMSO and 25% FBS were thawed, dissected into 1‐mm^3^ pieces and explanted onto gelatine dental sponges (Spongostan, Johnson & Johnson, Humanus Dental AB, *MEDEON Science Park*, SE, Sweden) in 24 well plates as described previously.^24^ Sponges were immersed in RPMI media supplemented with 10% FBS, PFS antibiotics (Sigma Aldrich), and complete X‐VIVO media with the following cytokines IL‐2 (50 U mL^−1^), IL‐7 (5 ng mL^−1^), and IL‐15 (0.5 ng mL^−1^). Explants were then treated with PBS, carboplatin (100 μmol L^−1^, Hospira Australia, Sydney, NSW  AUS), UT T cells (CD3 or 1:1 CD4:CD8 cells) or nfP2X7‐CAR‐T cells (CD3 or 1:1 CD4:CD8) in a humidified atmosphere at 37°C containing 5% CO_2_ for 48 h. Tissue explants were then fixed with 10% buffered formalin (Sigma Aldrich), processed for histology and immunohistochemistry. The clinical and pathological characteristics of the ovarian cancer patients used in the explant assays are summarised in Supplementary table 1.

### *In vivo* xenografts

OVCAR3‐Luc (5 × 10^6^) were prepared in a final volume of 250 μL in sterile 1× PBS and engrafted via intraperitoneal (i.p.) injection into 5–6‐week‐old female NOD‐*scid* IL2Rγ^null^ (NSG) mice (Animal Resource Centre, Perth, WA, Australia). CD3 T cells were thawed from frozen stocks stored in liquid nitrogen (Batchs 21 and 30) and washed in X‐VIVO media with cytokines IL‐2 (50 U mL^−1^), IL‐7 (5 ng mL^−1^), and IL‐15 (0.5 ng mL^−1^) and sterile PBS. 1 × 10^7^ UT CD3 cells or nfP2X7‐CAR‐T cells were injected i.p. into the mice (in a total volume of 250 μL) after 21 days (Experiment 1) or 7 days (Experiment 2) post‐OVCAR3 cell injection. Tumour growth was monitored using the IVIS imaging system and mice were followed for up to 160 days (Experiment 1) and 63 days (Experiment 2) post‐tumour cell injection. Prior to imaging, mice were injected i.p. with D‐luciferin mono potassium salt (30 mg mL^−1^, 100 μL/mouse, cat. #88294, Life Technologies). At experimental endpoint, tumour tissues were collected, fixed in 10% buffered formalin (Sigma Aldrich) and embedded in paraffin wax for histological analysis.

### Immunohistochemistry

Tissues sections were heated at 60°C for 90 min, followed by microwave antigen retrieval and blocking as described previously.^53^ Slides were incubated overnight at 4°C with rabbit polyclonal antibody to the P2X7 receptor, which detects both wild‐type and nfP2X7 (1/600, NBP2‐19654, Novus Biologicals, Centennial, CO, USA) or cleaved caspase‐3 as a measure of apoptosis (1/200, 9661L, Cell Signalling Technology, Danvers, MA) as described previously.^24^ T cells in explant tissues and OVCAR3 xenograft tumours were detected using CD3 (1/200, rabbit monoclonal antibody clone SP7, Abcam, Cambridge, MA, USA). OVCAR3 xenograft tumours additionally analysed by immunohistochemistry using antibodies to CD4 (1/200, mouse monoclonal, clone N1UG0, Invitrogen, Thermo Fisher), CD8 (1/200, rabbit monoclonal, clone SP16, Invitrogen, Thermo Fisher) and PD1 (1/200, rabbit monoclonal, clone D4W2J, Cell Signalling Technology). Following overnight incubation with the primary antibodies visualisation of immunoreactivity was achieved using biotinylated anti‐rabbit immunoglobulins (1/400, Dako Australia, Sydney, NSW, Australia) or mouse immunoglobulins (1/400, Dako Australia), followed by streptavidin‐HRP (1/500, Dako Australia) and diaminobenzene (Sigma Aldrich) as described previously.^53^ All slides were counterstained with haematoxylin, dehydrated, mounted in Pertex, and digitally scanned using NanoZoomer Digital Pathology System (Hamamatsu Photonics, SZK, Japan). For cleaved caspase 3 immunostaining, images (*n* = 10–15 images per treatment group) from NanoZoomer digital files were randomly selected across the tissue fragments and the number of positive cleaved caspase 3 positive cells were counted visually by 2 or 3 researchers who were blinded to the treatment. The average number of positive cleaved caspase cells/mm^2^ from two independent immunostaining runs was determined and expressed as % of control (PBS or UT T cells). The % positive CD3, CD4, CD8, PD1, and P2X7 cells in the explant tissues, OVCAR3 xenograft tumours or ovarian cancer tissues were measured using QuPath version 0.2.3 (35). A single threshold was used to measure positivity for CD3, CD4, CD8, and PD1. Three thresholds (weak, moderate and strong) were used to generate an H‐score between 0 and 300 for the P2X7 immunostaining. A high grade serous ovarian carcinoma (HGSOC) with immune cells was used as a positive control for CD3, CD4, CD8 and PD1 staining. Explant tissues previously treated with carboplatin were used as positive control for cleaved caspase 3. MDA‐MB231 tumour cells known to express nfP2X7 receptor were used as positive control for the P2X7 receptor immunostaining. Negative controls included no primary antibody or incubation with mouse or rabbit IgG.

### Statistical analysis

The Student's *t*‐test or one‐way ANOVA with Tukey's multiple comparison test was used to assess statistical significance between control and treatment groups (MTT and spheroid assays using GraphPad Prism Version 9 for Windows, GraphPad Software, La Jolla California USA). A paired Wilcoxon test was used to determine statistical significance between treatments in the explant assays (GraphPad Prism 9). For the *in vivo* mouse model experiments, the Student's *t‐*test (Experiment 1) or One‐way ANOVA with Tukey post‐hoc test for multiple comparisons (Experiment 2) were used to determine significance between control and treatment groups. The Mann–Whitney *U*‐test was used to assess difference in CD4, CD8 and PD1 positivity between control and treatment groups. Statistical significance was accepted at *P‐*value < 0.05.

## Author contributions

**Veronika Bandara:** Investigation; methodology; resources; writing – review and editing. **Victoria M Niktaras:** Data curation; formal analysis; investigation; methodology; writing – original draft; writing – review and editing. **Vasiliki J Willett:** Formal analysis; investigation; methodology; writing – original draft; writing – review and editing. **Hayley Chapman:** Formal analysis; investigation; methodology. **Noor A Lokman:** Methodology; project administration; supervision; writing – original draft; writing – review and editing. **Anne M Macpherson:** Methodology; project administration; resources; supervision; writing – original draft. **Silvana Napoli:** Methodology; resources. **Batjargal Gundsambuu:** Investigation; methodology; resources. **Jade Foeng:** Investigation; methodology; resources; writing – review and editing. **Timothy J Sadlon:** Methodology; writing – review and editing. **Justin Coombs:** Conceptualization; methodology; resources. **Shaun R McColl:** Conceptualization; methodology; resources; writing – review and editing. **Simon C Barry:** Conceptualization; funding acquisition; investigation; methodology; supervision; writing – original draft; writing – review and editing. **Martin K Oehler:** Conceptualization; funding acquisition; investigation; writing – review and editing. **Carmela Ricciardelli:** Conceptualization; funding acquisition; investigation; methodology; supervision; writing – original draft; writing – review and editing.

## Conflict of interest

The authors report no conflict of interest.

## Ethical approval and consent to participate

Primary ovarian cancer cells derived from ascites were collected from advanced stage HGSOC patients with informed patient consent and approval from the Central Adelaide Local Health Network Research Ethics Committee (CALHN Reference number R2018215). Tissue for explant assays were collected at surgery with informed patient consent and approval from CALHN Research Ethics Committee (CALHN Reference number R20180419). Animal experiments were performed with ethics approval from University of Adelaide Animal Ethics Committee (M‐2018‐125).

## Supporting information


Supplementary table 1
Supplementary table 2
Supplementary figure 1
Supplementary figure 2
Supplementary figure 3
Supplementary figure 4
Supplementary figure 5
Supplementary figure 6
Supplementary figure 7
Supplementary figure 8
Supplementary figure 9

## Data Availability

The data used and/or analysed during the current study are available from the corresponding author on reasonable request.
