# Supplementary material for: Engineered CAR‐T cells targeting the non‐functional P2X purinoceptor 7 (P2X7) receptor as a novel treatment for ovarian cancer
Source: Clin Transl Immunology. 2024 May 23;13(5):e1512. doi: 10.1002/cti2.1512 (PMC11116765; doi:10.1002/cti2.1512)
Supplement: Supplementary file 1 — Supplementary table 1 Supplementary table 2 Supplementary figure 1 Supplementary figure 2 Supplementary figure 3 Supplementary figure 4 Supplementary figure 5 Supplementary figure 6 Supplementary figure 7 Supplementary figure 8 Supplementary figure 9 [file CTI2-13-e1512-s001.docx]

**Supplementary data**

**Supplementary table 1**. Summary of patient information for primary ovarian cancer cells derived from ascites and tissues for the patient derived explant assay (PDE)

| Patient no. | Diagnosis | Clinical stage at Diagnosis | Tumor grade | Assay |
| --- | --- | --- | --- | --- |
| 1 | Serous carcinoma of the ovary | 3C | 3 | MTT |
| 2 | Serous carcinoma of the peritoneum | 3C | 3 | MTT, spheroid |
| 3 | Serous carcinoma of the peritoneum | 3C | 3 | MMT, spheroid |
| 4 | Serous carcinoma of the fallopian tube | 2B | 3 | PDE |
| 5 | Serous carcinoma of the peritoneum | 3C | 3 | MTT spheroid  PDE |
| 6 | Serous carcinoma of the ovary | 3C | 3 | PDE |
| 7 | Endometrioid carcinoma of the ovary | 2 | 2 | PDE |
| 8 | Serous carcinoma of the ovary | 2B | 3 | PDE |
| 9 | Serous carcinoma of the ovary | 1C | 3 | PDE |
| 10 | Serous carcinoma of the ovary | 2B | 3 | PDE |
| 11 | Serous carcinoma of the ovary | 3C | 3 | MTT spheroid  PDE |
| 12 | Serous carcinoma of the ovary | 1A | 3 | PDE |
| 13 | Serous carcinoma of the ovary | 2B | 3 | MTT spheroid  PDE |
| 14 | Serous carcinoma of the peritoneum | 3C | 3 | MTT |
| 15 | Serous carcinoma of the ovary | 3B | 3 | MTT |
| 16 | Recurrent serous carcinoma of the ovary | 1C | 3 | MTT |
| 17 | Serous carcinoma of the ovary | 4 | 3 | MTT |

MTT, 3-(4,5-dimethylthiazol-2-yl)-2,5-diphenyl-2H-tetrazolium bromide, PDE, patient derived explant

**Supplementary table 2.** Summary of T cell characteristics in the different batches of nfP2X7 CAR-T cells with different assays used in this study.

| Batch number | Experimental procedure | T cell subtype | Batch composition | EGFR (d8 post transfection) | PD1 |
| --- | --- | --- | --- | --- | --- |
| 7 | **PDE**: P5, P6, P7 | CD3  CD4/CD8 (1:1) | CD4^+^ (23%)  CD8^+^ (75%) | CD3^+^ (66%) CD4^+^ (84%)  CD8^+^ (72%) | CD4^+^ (1.6%)  CD8^+^ (33.6%) |
| 9 | **PDE**: P4, P5, P8, P10, P11, P12 | CD4/CD8 (1:1) |  | CD4+ (59%)  CD8+ (48%) | N/A |
| 11 | **PDE**: P8, P9, P13 | CD3 &  CD4/CD8  (1:1) | CD4^+^ (24%) CD8^+^ (75%) | CD3^+^ (58%)  CD4^+^ (68%)  CD8^+^ (75%) | N/A |
| 13 | **Spheroid**:OVCAR3, SKOV3, OVCAR5. | CD3  &  CD4/CD8 (1:1) | CD4^+^ 69.9%)  CD8^+^ (30.1%) | CD3^+^ (57%)  CD4^+^ (67%)  CD8^+^ (42%) | CD4^+^ (18.4%)  CD8^+^ (6.42%) |
| 15 | **MTT**:OVCAR3, OV90, P3, P5, P13  **Spheroid**:OVCAR3, OVCAR5, P3, P5, P11, P13. | CD3  CD3 | CD4 (14.3%)  CD8 (85.7%) | CD3 (63%)  CD4 (64%)  CD8 (54%) | CD4^+^ (67.9%)  CD8^+^ (53.4%) |
| 16 | **MTT:** OVCAR3, SKOV3, OVCAR5, P1, P2, P4, P5, P6  **Spheroid**:OVCAR3, SKOV3, P2, P3, P5, P11  **PDE**: P6, P7, P9, P10, P11, P12, P13 | CD3  CD3  CD3 & CD4/CD8 (1:1) | CD4^+^ (22.6%)  CD8^+^ (77.4%) | CD3^+^ (59%)  CD4^+^ (84%)  CD8^+^ (52%) | CD4^+^ (1.10%)  CD8^+^ (1.16%) |
| 18 | **MTT**: OVCAR3, SKOV3, OVCAR5, LP9 | CD3 | n/a | CD3^+^ (49%)  CD4^+^ (46%)  CD8^+^ (39%) | n/a |
| 21 | **MTT**: OVCAR3, SKOV3, OVCAR5, LP9, P11, P14 | CD3 | CD4^+^ (49.8%)  CD8^+^ (50.2%) | CD3^+^ (67%)  CD4^+^ (70%)  CD8^+^ (65%) | CD4^+^ (35.8%)  CD8^+^ (24.4%) |
| 21 | OVCAR3 xenograft (experiment 1) | CD3  (post thawing) | CD4^+^ (48.2%)  CD8^+^ (50.5% | CD3^+^ (63.5%)  CD4^+^ (65%)  CD8^+^ (64%) | CD4^+^ (69.2%)  CD8^+^ (68.6%) |
| 25 | **MTT:** OVCAR5, OVCAR3, P11, P14, P15, P16 | CD3 | CD4+ (65.7%)  CD8+ (30.7%) | CD3^+^ (65.1%)  CD4^+^ (66.9%)  CD8^+^ (70.3%) | CD4^+^ (82.2%)  CD8^+^ (54.8%) |
| 26 | **MTT:** OVCAR5**,** P16, P17 | CD3 | CD4+ (38.5%)  CD8+ (60.1%) | CD3^+^ (20%)  CD4^+^ (33%)  CD8^+^ (30.4%) | CD4^+^ (16.3%)  CD8^+^ (0.36%) |
| 27 | **MTT:** OVCAR3, OVCAR5, P11, P15, P16 | CD3 | CD4^+^ (86.8%)  CD8^+^ (11.6%) | CD3^+^ (88.8%)  CD4^+^ (86.1%)  CD8^+^ (100%) | CD4^+^ (32.7%)  CD8^+^ (25.6%) |
| 30 | **MTT**: OVCAR3, P16  OVCAR3 xenograft (experiment 2) | CD3  (D19 at storage) | CD4^+^ (23.4%)  CD8^+^ (75.3%) | CD3^+^ (50.9%)  CD4^+^ (45%)  CD8^+^ (55.3%) | CD4^+^ (76.0%)  CD8^+^ (5.7%) |
| 31 | **MTT& IFNγ assay**: OVCAR3, SKOV3, LP9 cells | CD3 | CD4^+^ (48.4%)  CD8^+^ (45.3%) | CD3^+^ (67.8%)  CD4^+^ (68.7%)  CD8^+^ (67.4%) | CD4^+^ (10.2%)  CD8^+^ (4.19%) |
| 32 | **MTT & IFNγ assay**: OVCAR3, SKOV3, LP9 cells P3, P5, P11, P15, P16, P17 | CD3 | CD4^+^ (49.6%)  CD8^+^ (44.9%) | CD3^+^ (73.7%)  CD4^+^ (87.5%)  CD8^+^ (58.3%) | CD4^+^ (1.31%)  CD8^+^ (0.14%) |
| 33 | **MTT & IFNγ assay**: OVCAR3, SKOV3, LP9 cells | CD3 | CD4^+^ (72.5%)  CD8^+^ (23.1%) | CD3^+^ (84.1%)  CD4^+^ (82.0%)  CD8^+^ (98.0%) | CD4^+^ (5.04%)  CD8^+^ (1.11%) |

PDE, patient derived explants; P, patient number for primary ovarian cells, P1-17


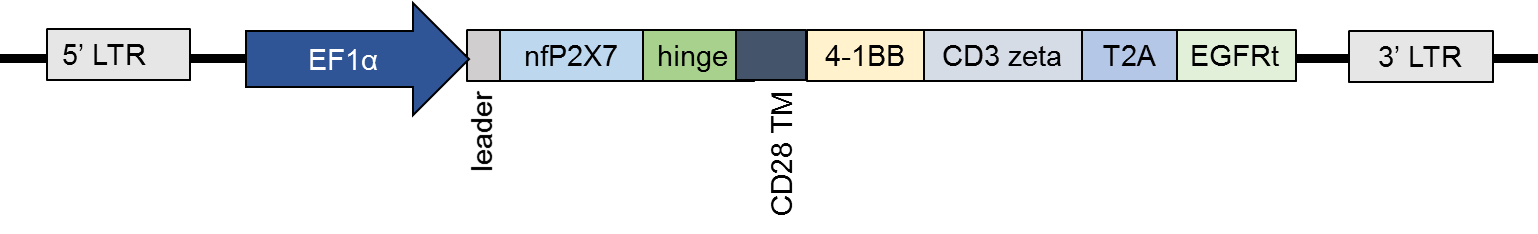


**Supplementary figure 1.** **nfP2X7-CAR-T lentivirus construct design**. The nfP2X7-CAR lentiviral construct harbours an EF1α promoter, leader sequence, nfP2X7 binding domain (scFv), linker sequence, CD28 transmembrane domain and CD3ζ and 4-1BB co-stimulation domains, which are co-expressed with a truncated EGFR sequence as described previously. ^21^

(a) (b)

**Supplementary figure 2**. **Effects of nfP2X7 CAR-T cells using the BrightGlo Luciferase *in vitro* cytotoxicity assay**. OVCAR3 and OVCAR5 cells treated with CD3 chimeric antigen receptor T (CAR-T, orange lines) cells or un-transduced (UT, blue lines) CD3 cells at effector:target ratio (E:T ratio, 10:1, 3:1 or 1:1) for 18–24 h **(a)** OVCAR5 and **(b)** OVCAR3. Data are the mean ± SEM from 3 independent experiments. *** *P-*value < 0.001, *** *P-*value < 0.0001, Student’s *t*-test.


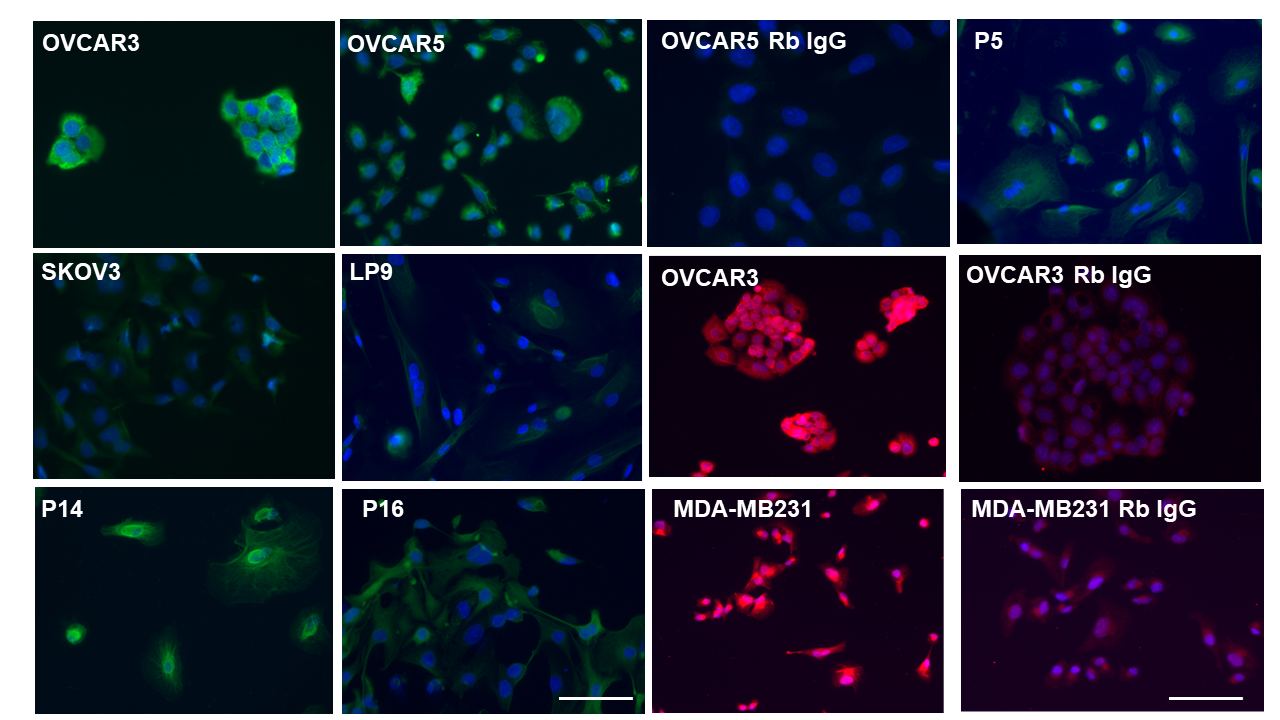


**Supplementary figure 3**: **P2X7 receptor expression in human ovarian cancer cells**. P2X7 receptor immunocytochemistry using rabbit polyclonal P2X7 antibody (1/200, Novus NBP2-19654). Images for ovarian cancer cell lines (OVCAR3, SKOV3, OVCAR5, LP-9 (normal peritoneal cells) and primary ovarian cancer cells (P5, P14 & P16) using secondary goat anti-rabbit-Alexa Fluor ® 488(1/200, Molecular Probes, Life Technologies. Images for OVCAR3 and breast cancer cell line (MDA-MB231) shown using secondary goat anti-rabbit-Alexa Fluor ® 594 (1/200, Molecular Probes, Life Technologies). Rabbit IgG (4 µg mL^–1^) controls shown for OVCAR5, OVCAR3 and MDA-MB231 cells. All images are the same magnification. Scale bar = 50 µm. Blue = DAPI, Green = P2X7 receptor (Alexa-488) or red P2X7 receptor (Alexa-594)


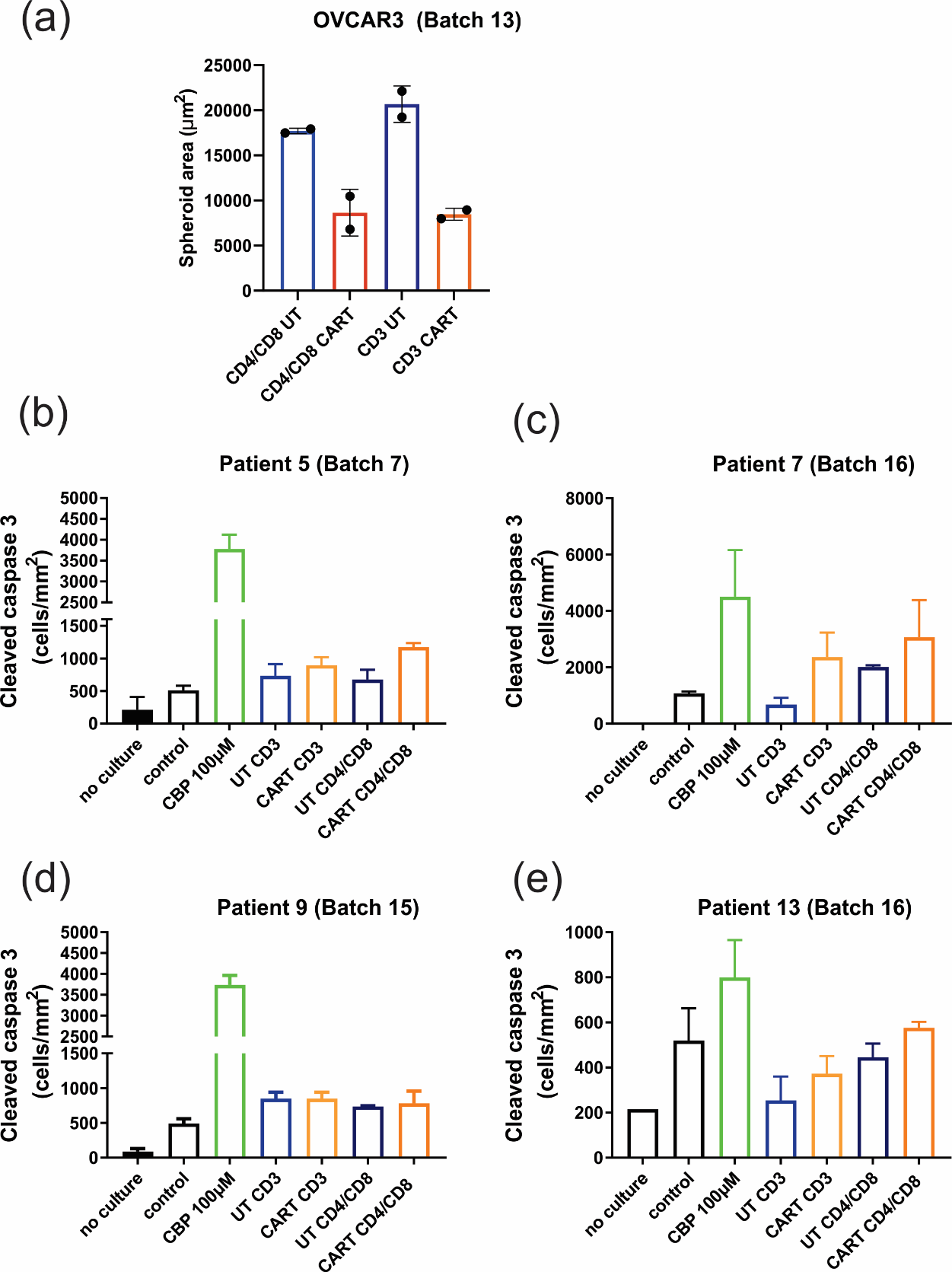


**Supplementary figure 4.** **Comparison of the effects between unselected CD3^+^ or a 1:1 mixture of CD4:CD8 nfP2X7 CAR-T cells**. **(a)**. spheroid assay and patient-derived explant (PDE) assays **(b–e**). Carboplatin (CBP). Data in **(a**) are duplicates from 1 CAR-T preparation (batch 13) and data in **(b–d**) are from a single experiment using 3 different CAR-T preparations (batch 7, batchs 15 &16). All treatments were for 48 h. Errors bars are mean ± SEM from two independent immunostaining runs (except no culture control for patient 13). No culture is uncultured tissue fixed prior to setting up the PDE assay and control is PBS treatment (carboplatin vehicle control).

**
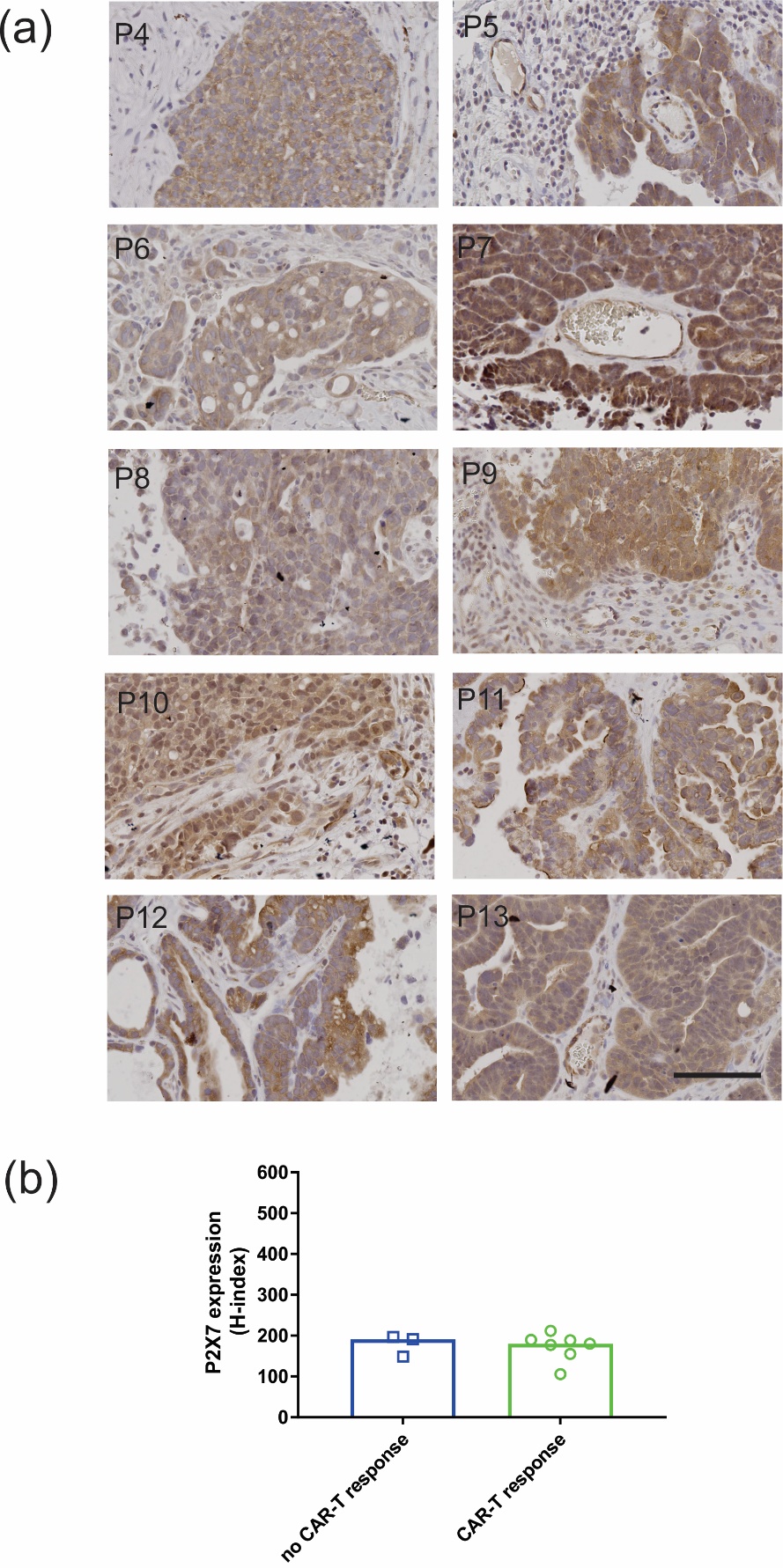
**

**Supplementary figure 5.** **P2X7 expression in patient ovarian cancer tissues used in patient-derived explant assays**. **(a)**. P2X7 receptor immunohistochemistry. Scale bar = 100µm. All images are the same magnification. **(b)**. Quantitation of P2X7 receptor expression using QuPath. H-index in patient tissues that showed no response (n = 3) or response to treatment with nfPX27 CAR-T cells (n = 7). Data are average H-score from 5–10 images for each tissue.


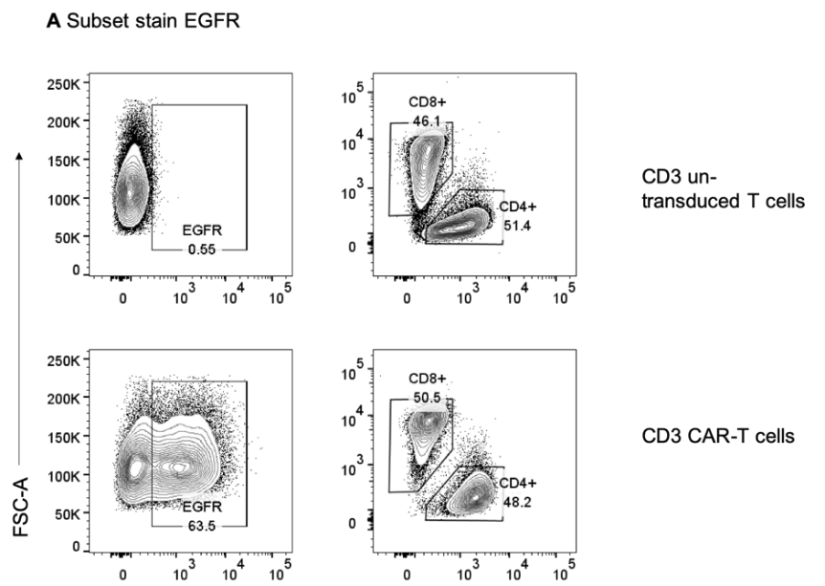

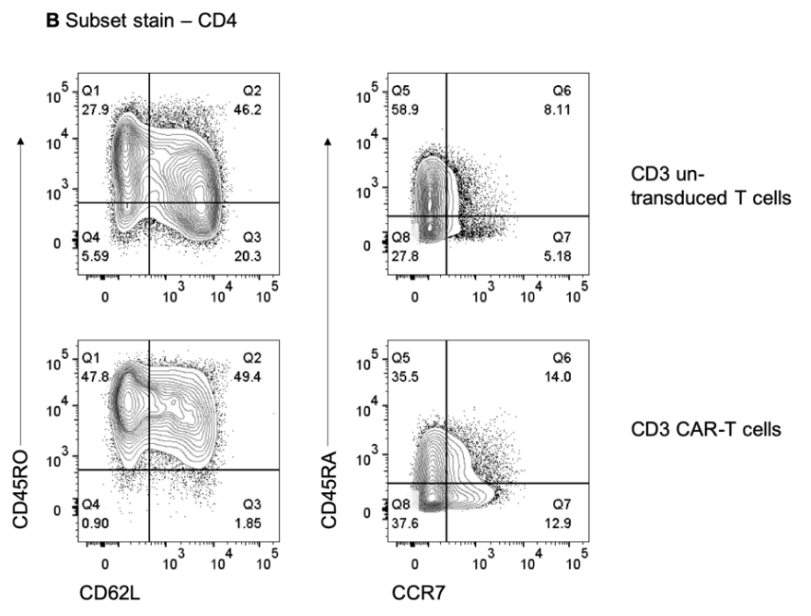


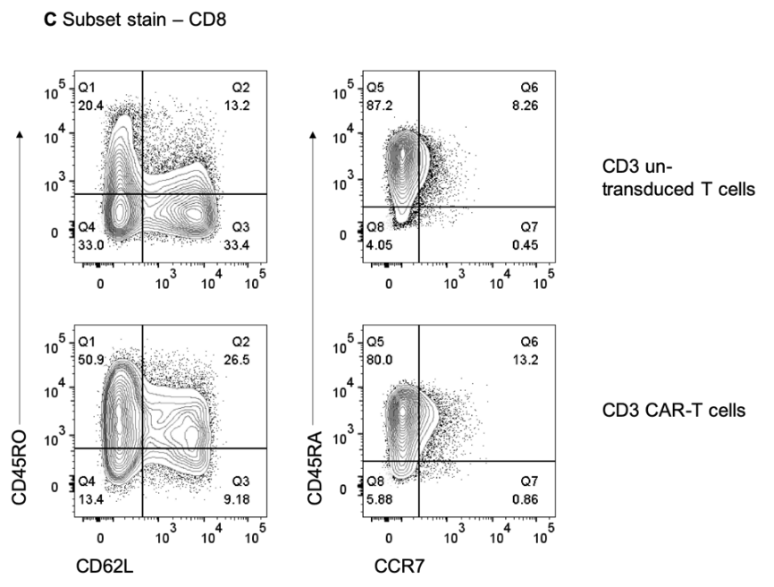


**Supplementary figure 6.** **Expression of EGFR, CD4 and CD8 by CAR-T cells**. nfP2X7-targeting CAR-T cells (batch 21) were generated and frozen at D12 post-PBMC before thawing for *in vivo* delivery and flow cytometric analysis. Representative FACS plots of **(a)** EGFR expression and CD4^+^/CD8^+^ ratio, pre-gated on CD3^+^ T cells for un-transduced (UT) and nfP2X_7_ CAR-T cells. Representative FACS plots of T cell subset phenotype of **(b)** CD4 and **(b)** CD8 T cells. CAR-T cells shown are from Batch #21.


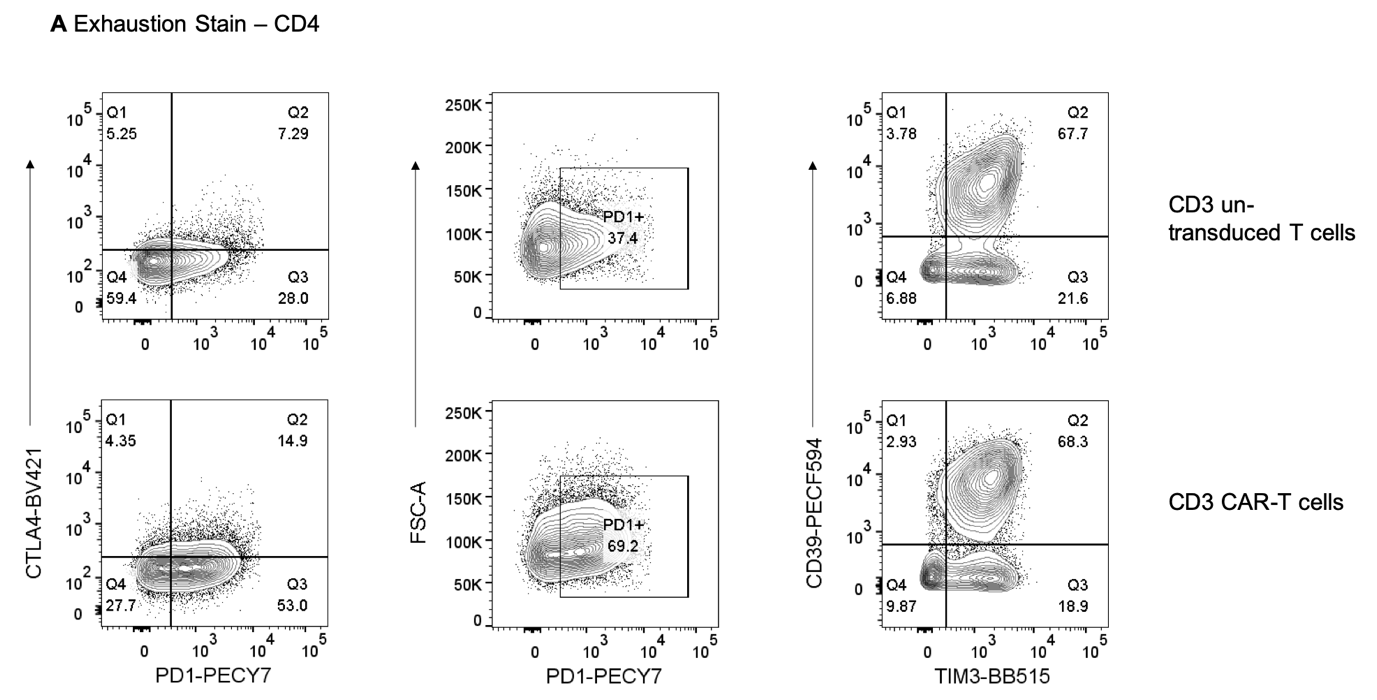


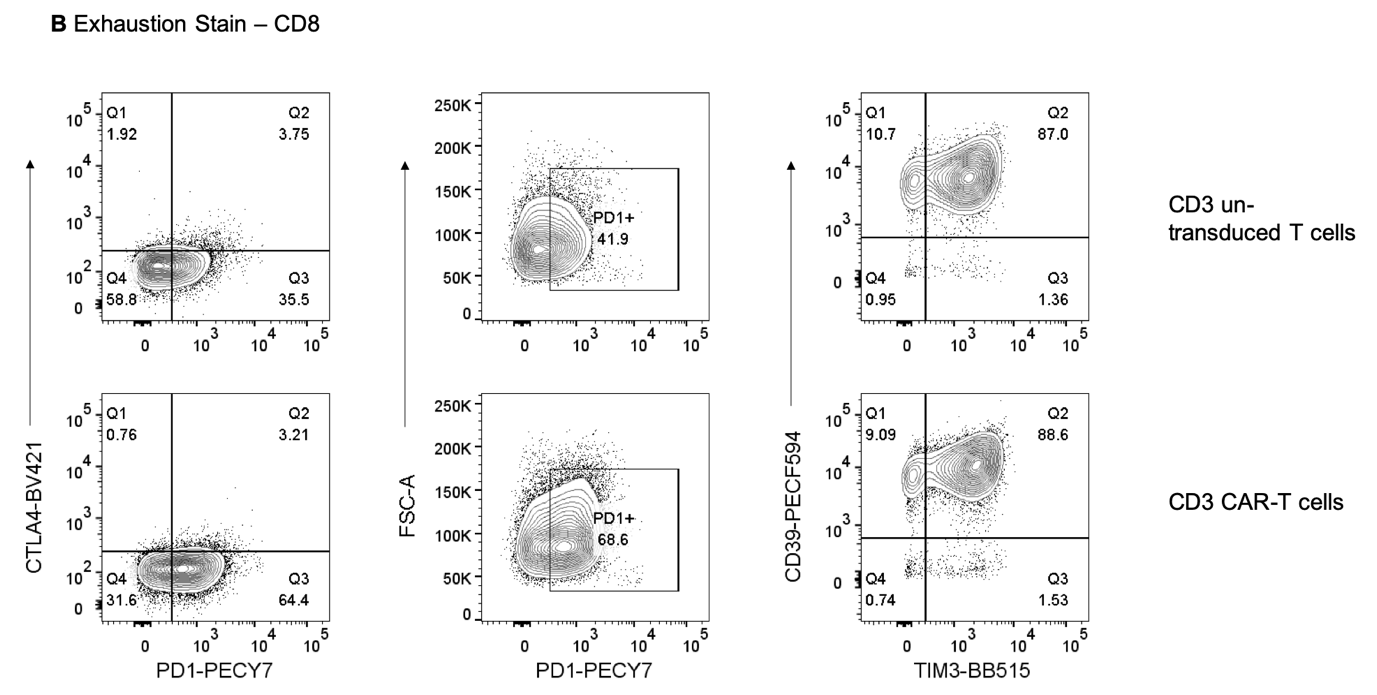


**Supplementary figure 7: Expression of co-inhibitory receptors and molecules by CAR-T cells.** nfP2X7-targeting CAR-T cells were generated and frozen at D12 post-PBMC before thawing for *in vivo* delivery and flow cytometric analysis. Representative FACS plots of PD1, CTLA-4, CD39 and TIM-3 expression of **(a)** CD4 and **(b)** CD8 T cells. CAR-T cells shown are from Batch #21.

**
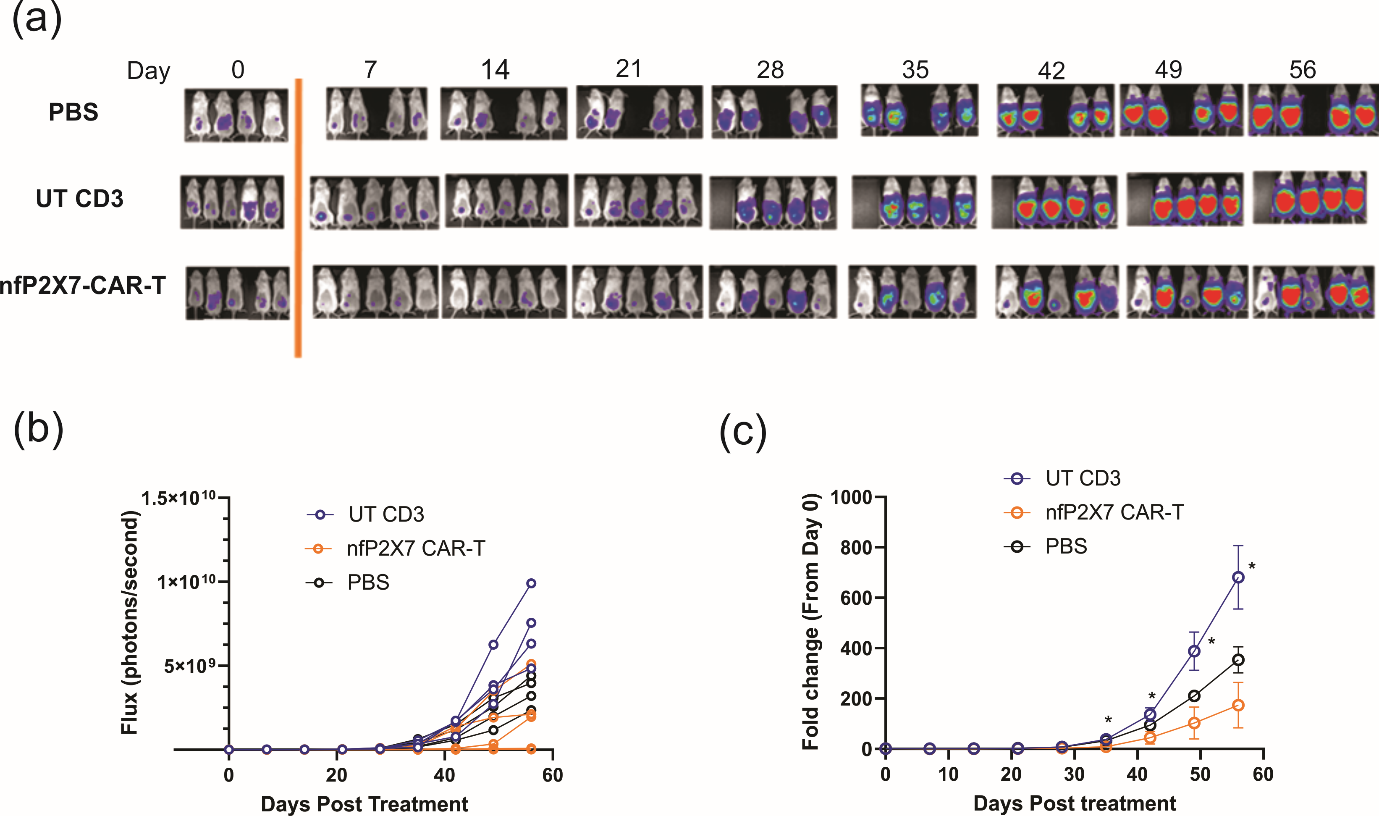
**

**Supplementary figure 8: Effects of intraperitoneal (i.p.) delivery of nfP2X7 CAR-T cells (Batch 30) on ovarian cancer metastasis in OVCAR3-luc xenografts (Experiment 2)**. **(a)** Bioluminescence flux imaging from OVCAR3-luc tumor bearing NSG mice injected with 2x10^6^ cells and treated i.p. with PBS (n = 4), 1x10^7^ cells un-transduced (UT) CD3 cells (n = 5) or nfP2X7-CAR-T (n = 5) cells on day 7 post-tumor injection (day 0). Imaging was performed between days 0 and 56 post-tumor injection. **(b)** Quantification of flux of each OVCAR3-luc bearing mouse treated i.p. with PBS, CD3 UT cells (n = 5) or nfP2X7-CAR-T cells (n=5). **(c)** Average fold change in Flux (from day 0) of OVCAR3-luc bearing mice. i.p delivery of nfP2X7 CD3 CAR-T cells at day 0 significantly reduced tumor burden in OVCAR3-luc tumor-bearing mice, compared with UT CD3 cells at day 28, 35, 42 & 56. Error bars mean ± SEM, * *P-*value < 0.05, comparisons at each time point, One-way ANOVA, Tukey Multiple comparison test.

**
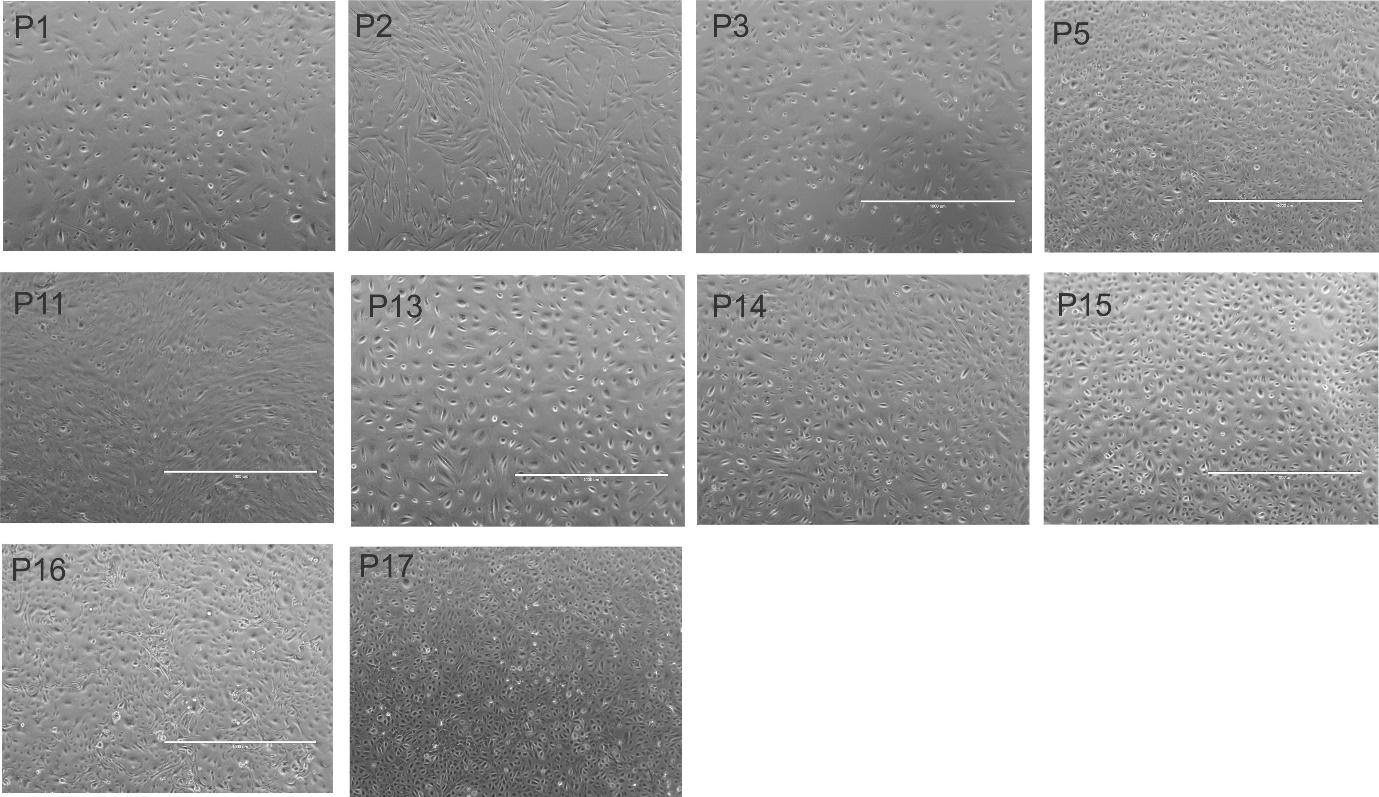
**

**Supplementary figure 9**. **Primary cells in monolayer culture.** Images of primary ovarian cancer cells derived from ascites prior to plating for the MTT or spheroid assays. All primary cell lines are between P1 and P3 passages, thawed from liquid nitrogen storage. Scale bar = 1000 µm.
